# Schizophrenia Impacts and Treatment Priorities: Results of An Externally-Led Patient-Focused Drug Development Meeting Survey

**DOI:** 10.1007/s43441-026-01001-2

**Published:** 2026-07-23

**Authors:** Ilse S. Peterson, Ramaa Chitale, Theresa Frangiosa, Holly B. Krasa, Paulie VonEdWærd-Benjamin, Anita Fisher, Jessica T. Markowitz, Arun Nagendra

**Affiliations:** 1 Faegre Drinker Consulting, 1500 K Street NW, Suite 1100, Washington, DC 20005 USA; 2 Subcontractor to Faegre Drinker Consulting, Washington, DC United States; 3 Schizophrenia & Psychosis Action Alliance, Alexandria, VA USA; 4 Blue Persimmon Group, Washington, DC USA; 5 PFDD Meeting Co-Moderator & Diagnosee, Michigan, USA; 6 Fisher Mental Health Consulting, Spring Valley, CA USA

**Keywords:** Schizophrenia, Patient-focused drug development, PFDD, Treatment needs

## Abstract

**Objective:**

Using survey data gathered for an Externally-Led Patient-Focused Drug Development (EL-PFDD) meeting led by the schizophrenia community to inform the US Food and Drug Administration, this publication examines schizophrenia’s impacts on daily life, reasons for discontinuation of medication, and desired outcomes of future medications.

**Methods:**

An online, cross-sectional survey about schizophrenia’s impacts on daily life, treatment experiences, and desired outcomes of future medications was developed for adults with schizophrenia and caregivers of adults with schizophrenia who could not complete the survey. Patient advocacy organizations participating in the EL-PFDD meeting recruited respondents via email and social media between August 9, 2022 and September 26, 2022.

**Results:**

282 survey responses from 96 people with schizophrenia and 186 caregiver proxies are reflected in this publication. Both respondent groups reported significant impacts of schizophrenia on aspects of daily living spanning work and education, relationships, responsibilities at home, and finances. For people with schizophrenia, the most frequently reported reason for discontinuing medications was weight gain (66% of 83 question respondents); for caregiver proxies, it was “feeling flat” (57% of 165 question respondents). Preventing delusions, preventing hallucinations, and enjoying daily activities received the highest average ratings as desired outcomes of future treatments from people with schizophrenia, whereas preventing delusions and making it easier to maintain relationships had the highest average ratings from caregiver proxies.

**Conclusions:**

As exploratory work on community treatment priorities, this research can inform patient-focused drug development endeavors by patients, advocates, industry, and regulators to address unmet needs in schizophrenia moving forward.

**Supplementary Information:**

The online version contains supplementary material available at https://doi.org/10.1007/s43441-026-01001-2.

## Introduction

Patient-focused drug development (PFDD) incorporates the experiences, perspectives, needs, and priorities of people with lived experience of diseases into medication development and regulatory decision-making [1]. PFDD has been formalized in the United States through enactment of the fifth Prescription Drug User Fee Act in 2012 and the 21st Century Cures Act in 2016. These statutes led to several new avenues for gathering and utilizing patient and caregiver input in drug development, including meetings that allow people with lived experience to share insights on symptom impacts and treatment needs directly with the US Food and Drug Administration (FDA).

PFDD’s emphasis on using lived experience to guide medication development is particularly relevant in schizophrenia, as approved FDA treatments have undertreated the diverse needs and challenges faced by individuals living with the disorder, resulting in limited treatment effectiveness and low adherence rates, which have been associated with drug efficacy and tolerability and a range of individual factors. [2] Innovation in schizophrenia medication development has been inhibited by poor understanding of the pathophysiology of schizophrenia, approaching schizophrenia spectrum disorders as a single diagnostic entity, and a focus on dopaminergic antagonism [3–5]. Medication adherence and discontinuation are common challenges, with estimates for non-adherence ranging from 40 to 50% [2, 6]. Moreover, symptoms that are major sources of disability—including cognitive and negative symptoms—are under-addressed by current medications, leading to low rates of functional recovery [3]. Importantly, recent advances in understanding the neurobiology of schizophrenia are driving the development of novel treatments, including those targeting glutamatergic pathways, inflammatory processes, and other non-dopaminergic systems [4, 7]. These innovations hold promise for addressing a broader range of symptoms with fewer side effects, which may lead to higher adherence rates.

This resurgence of innovation in schizophrenia treatments presents an opportune moment to integrate PFDD principles into medication development, ensuring that new therapies reflect the needs and preferences of those most affected by schizophrenia. PFDD’s formal recognition by regulatory agencies, including the FDA, ensures that patient insights can influence decision-making and underscores the importance of real-world and patient-reported outcomes. This will ultimately lead to therapies that have a meaningful impact on patients’ lives.

On November 2nd, 2022, the US schizophrenia community held an Externally-Led PFDD (EL-PFDD) meeting with the FDA, co-hosted by The American Foundation for Suicide Prevention (AFSP), Mental Health America (MHA), the National Alliance on Mental Illness (NAMI), the National Council for Mental Wellbeing, and the Schizophrenia and Psychosis Action Alliance (S&PAA) with support from Faegre Drinker Consulting. This meeting was an opportunity for people directly affected by schizophrenia to convey to the FDA the devastating effects of the disease and challenges with existing medications. Key takeaways from this meeting were published in 2023 in a Voice of the Patient (VoP) Report [8].

In tandem with this EL-PFDD meeting, S&PAA commissioned a cross-sectional survey to better understand day-to-day experiences of those with schizophrenia and aspects of the disease the schizophrenia community would like to see addressed by those developing medications. The survey served two purposes: informing EL-PFDD meeting planning and obtaining insight from a large population in a more comprehensive and systematic manner than would have been possible at the meeting. This manuscript presents data from S&PAA’s cross-sectional EL-PFDD meeting survey. Findings from this exploratory research are intended to contribute to a better understanding of the daily challenges of people with schizophrenia and to inform future drug development and evaluation, recognizing longstanding challenges in innovation surrounding schizophrenia treatment as well as the more recent emergence of novel medications.

## Methods

### Survey Development

An online survey was developed in the spring of 2022 and designed to elicit information on topics recommended for EL-PFDD meetings, including the frequency, severity, and significance of symptoms experienced by people with schizophrenia; how schizophrenia symptoms impact daily life; treatment and management approaches; why people do or do not use drug treatments; and desired outcomes of future drug treatments [9]. Survey creation was informed by the team’s knowledge and experience related to the FDA’s PFDD program goals and guidance documents, peer-reviewed literature and practice guidelines, and knowledge of and experience with drug development and evaluation processes [10–25]. The tool—included in the online supplement—included a combination of close-ended and open-ended questions and was built on the survey platform Alchemer.

Prior to its finalization, the survey was shared for review by S&PAA’s meeting partners, an S&PAA-affiliated group of people with schizophrenia and their caregivers, schizophrenia researchers, and two psychiatrists with extensive experience in schizophrenia. Input from these individuals and organizations guided revision and refinement of the survey questions and additional areas requiring clarification preceding submission to the Advarra IRB for an exemption determination, which was granted (Pro00064894).

### Eligibility Criteria, Informed Consent, and Recruitment

Individuals eligible to complete the survey included (i) adults with a diagnosis of schizophrenia who were diagnosed at least six months ago and (ii) caregivers of adults with schizophrenia who could not complete this survey on their own. It was not intended for professional, paid caregivers. Respondents were required to live in the United States and have reached the age of majority in their state or territory. All individuals interested in completing the survey completed an informed consent form, which is included at the beginning of the survey in the online supplement.

Participants were recruited via outreach by S&PAA, AFSP, MHA, NAMI, and the National Council for Mental Wellbeing. These organizations sent email announcements to their mailing lists and shared the survey on social media. The survey was open from August 9, 2022 to September 26, 2022.

### Data Cleaning

Stata/IC 16 was used for data cleaning. All partial and complete surveys were assessed to confirm that survey respondents met the eligibility criteria and that responses were not duplicates. Partial surveys were retained if respondents had completed at least one question within each of the topical sections of the survey. In two instances, a person with schizophrenia and one of their caregivers both completed surveys. In these cases, only the surveys from the affected individuals were retained so that the final dataset would reflect unique and independent observations.

### Descriptive Analyses

After data cleaning, the survey platform Alchemer, Stata, and Excel were used to generate tables and charts of all quantitative data. Data were stratified by respondent group (self-completed vs caregiver-completed) for two reasons: (i) the demographic characteristics of individuals represented within each category were different between the two groups, and (ii) caregivers may have given different answers than what would have been reported by the people for whom they care, despite being asked the questions as a proxy reporter.

The stratified data were jointly reviewed and interpreted by the author team. The survey questions deemed to be most relevant to the manuscript objectives were selected for presentation in this publication. Descriptive statistics are presented; because this was exploratory research rather than hypothesis-testing and because of the limitations posed by the self-reported nature of the data, additional statistical testing or regression modeling was not conducted.

### Qualitative Analysis

The survey included the open-ended question: “What have been the most significant impacts of schizophrenia on your life?” Responses to this question were coded using a hybrid deductive and inductive coding approach. Using a shared Excel workbook, two coders independently coded a sample of 100 responses, compared their codes for agreement, and finalized a codebook (see the online supplement). One coder then applied this codebook to the full sample. A second coder reviewed the dataset to confirm agreement. Disagreements were discussed and resolved with a third researcher. Excel was used to tabulate frequencies and percentages for the final codes.

## Results

### Participant Characteristics

A total of N = 282 people with schizophrenia were represented in the survey, 96 by self-completed surveys and 186 via caregiver-completed surveys (Table 1). The majority of people represented were taking at least one medication for psychiatric symptoms. Among those represented by self-completed surveys who provided information about current medications for schizophrenia (n = 94), 80.9% reported taking an oral antipsychotic, 17.0% reported taking a long-acting injectable antipsychotic, and 45.7% reported taking other psychiatric medications such as antidepressants and anxiolytics. For individuals represented by caregiver proxy surveys (n = 176), respective percentages were 76.7%, 23.3%, and 36.4%.

**Table 1:** Characteristics of people with schizophrenia represented in survey sample (N = 282)

Characteristics	Self-completed surveys		Caregiver-completed surveys	
	n = 96		n = 186	
	n	%	n	%
*Age*				
18–34	22	23	120	65
35–49	39	41	37	20
50–64	23	24	25	13
65 +	11	11	4	2
Unknown	1	1		
*Gender*				
Male	45	47	133	72
Female	44	46	36	19
Another identity	5	5	3	2
Prefer not to answer or unknown	2	2	14	8
*Race*				
American Indian or Alaska Native	1	1	1	1
Asian	8	8	3	2
Black or African American	3	3	5	3
White	71	74	150	81
Another Identity (Includes multiple races)	11	12	14	8
Unknown	2	2	13	7
*Ethnicity*				
Hispanic, Latino, or of Spanish origin	7	8	16	9
*Employment status*				
Full-time	21	22	9	5
Part-time	19	20	21	11
Actively looking	7	7	2	1
Not employed	32	33	151	81
Retired	13	14	1	1
Prefer not to say	4	4	2	1
*Educational status*				
Yes (full-time or part-time)	14	15	21	11
Not in school	82	85	164	88
Don't know	0	0	1	1
*Current living situation*				
A household with relatives or friends	56	58	125	67
Living independently, alone	30	31	18	10
Other housing*	8	8	34	18
Currently without housing	0	0	5	3
Unknown	2	2	4	2
*Insurance coverage*				
Any insurance	87	91	165	91
None	3	3	7	4
Prefer not to answer or unknown	6	6	14	8

*Includes supervised group housing, residential substance use programs, long-term care, correctional facility, shelter. Combined due to sample sizes

### Impacts of Schizophrenia on Daily Life

Across both respondent groups, negative symptoms were reported to have more significant impacts than positive symptoms (Fig. 1 and supplemental Table 1). Both those with schizophrenia and caregivers reported significant impacts of schizophrenia, particularly in the areas of work performance and relationships (Fig. 2 and supplemental Table 2). Caregiver proxies consistently rated the impact of schizophrenia on daily life as more severe compared to self-reported responses.

**Fig. 1:**
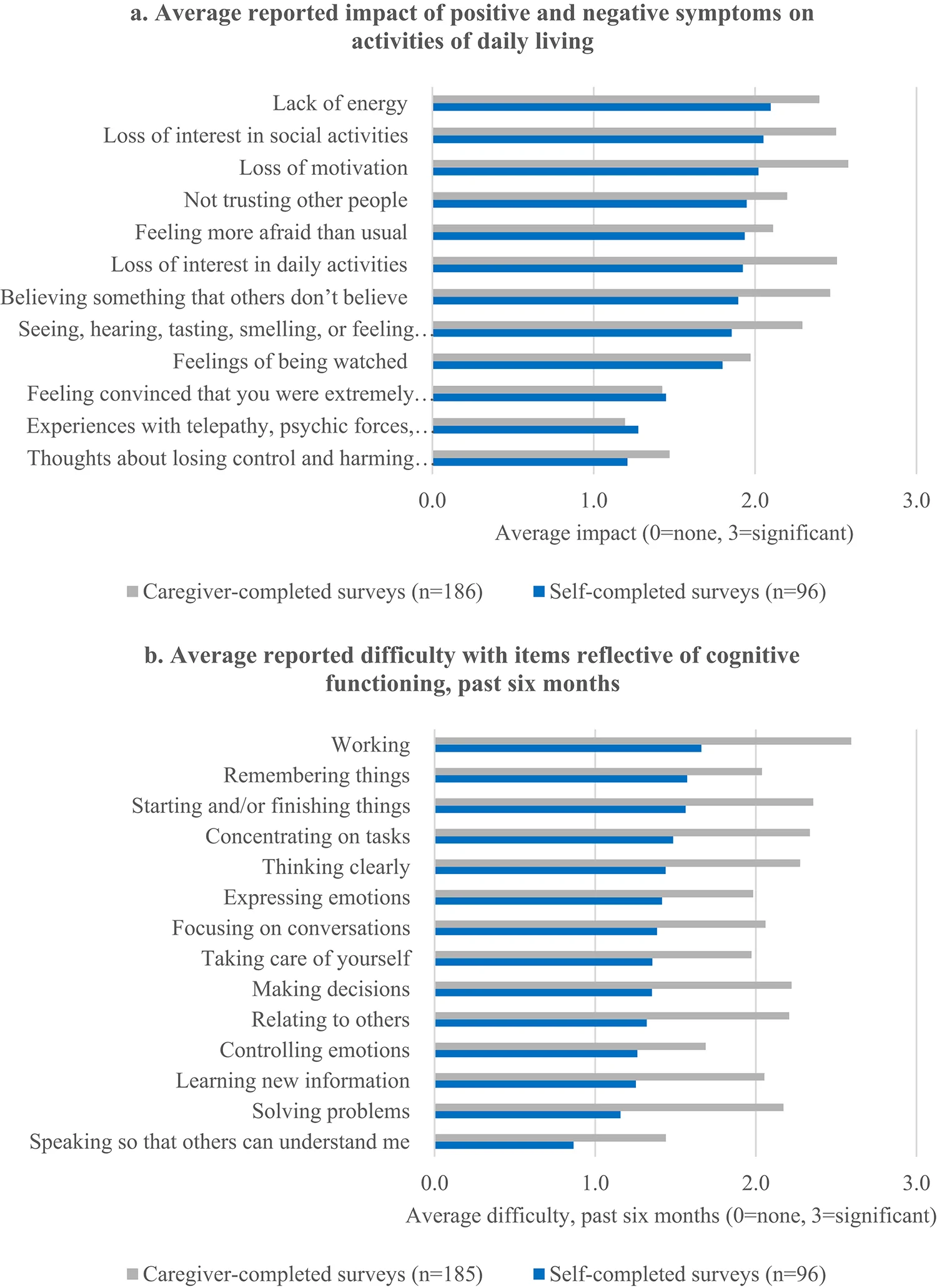
Impacts of positive, negative, and cognitive symptoms on daily life. Survey questions: **a** Schizophrenia may make it difficult to think, act, or live the way one wants. Have any of the following diminished your ability to function and live the way you would like? *Caregiver Version:* Schizophrenia may make it difficult to think, act, or live the way one wants. Have any of the following diminished the ability of the person you care for to function and live the way they would like? **b** In the past six months, have you had difficulty with any of the following? *Caregiver Version:* In the past six months, has the person you care for had difficulty with any of the following?

**Fig. 2:**
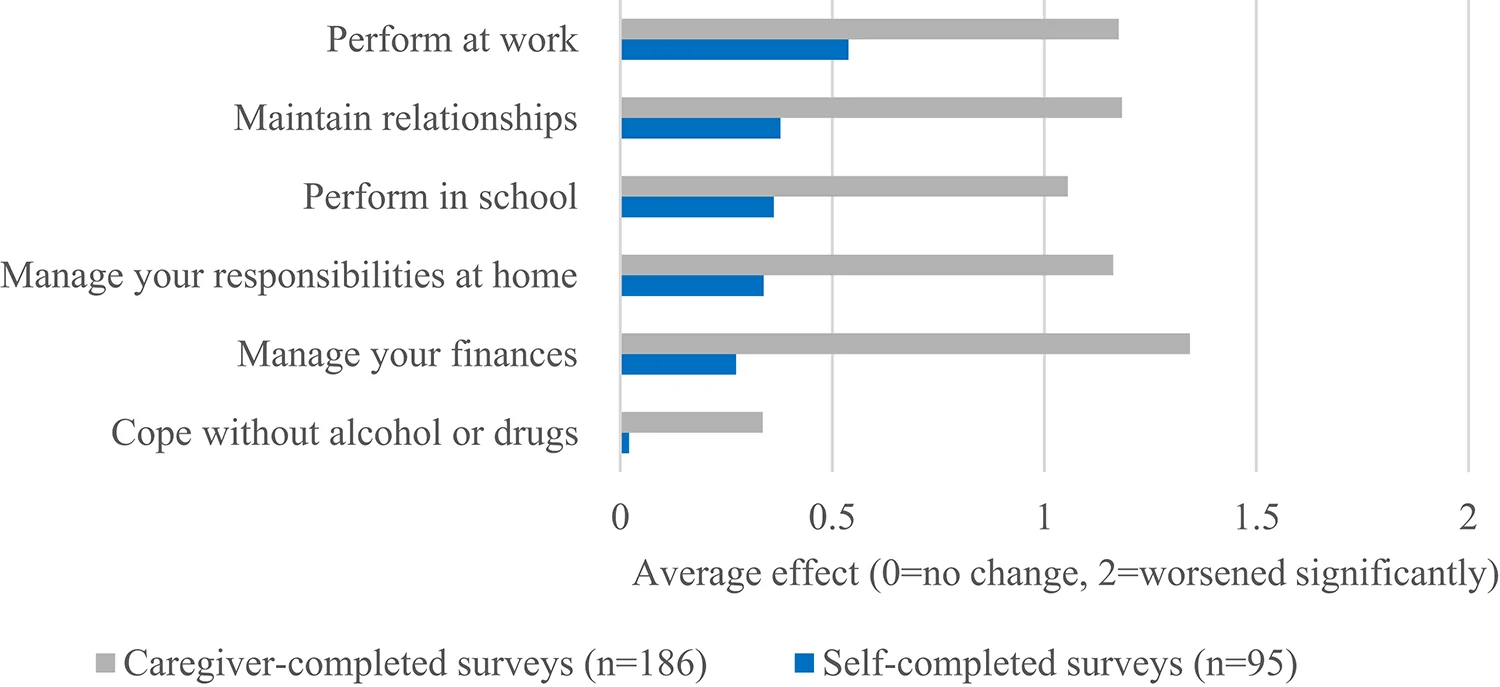
Effect of schizophrenia on ability to perform key aspects of daily living. Survey questions: Since you first started experiencing symptoms of schizophrenia, has your ability to do the following changed? *Caregiver Version:* Since the person you care for first started experiencing symptoms of schizophrenia, has their ability to do the following changed?

Two hundred forty-seven responses to the open-ended question about the impacts of schizophrenia on life spoke to disease impacts—including 71 responses from people with schizophrenia and 176 responses from caregiver proxies (supplemental Tables 3 and 4). Emergent themes included lost opportunities, concerns about the safety of oneself or others, and impacts on social functioning. The most frequently occurring code within lost opportunities related to vocational performance (with jobs or schooling), which was raised by 42% (n = 30) of respondents with schizophrenia and 45% (n = 80) of caregiver respondents. Within safety-related concerns, 8% (n = 6) of respondents with schizophrenia and 10% of caregivers (n = 18) described harm to the person they cared for, others, or property. Within social functioning, 42% (n = 30) of respondents with schizophrenia and 58% (n = 102) of caregiver proxies described a loss of friends, isolation, social withdrawal, and loss of socialization. Thirteen percent (n = 9) of respondents with schizophrenia and 14% (n = 24) of caregiver proxies said that the condition impacted “everything.”

### Challenges with Existing Treatments & Desired Attributes of Future Treatments

Weight gain, movement effects, and feeling flat were identified by both those with schizophrenia and caregivers as the most frequent reasons for quitting medications (Fig. 3 and supplemental Table 5). Preventing delusions was a top priority for future treatments among both respondent groups (Fig. 4 and supplemental Table 6). Caregiver proxy-completed surveys weighted several items more highly than people with schizophrenia.

**Fig. 3:**
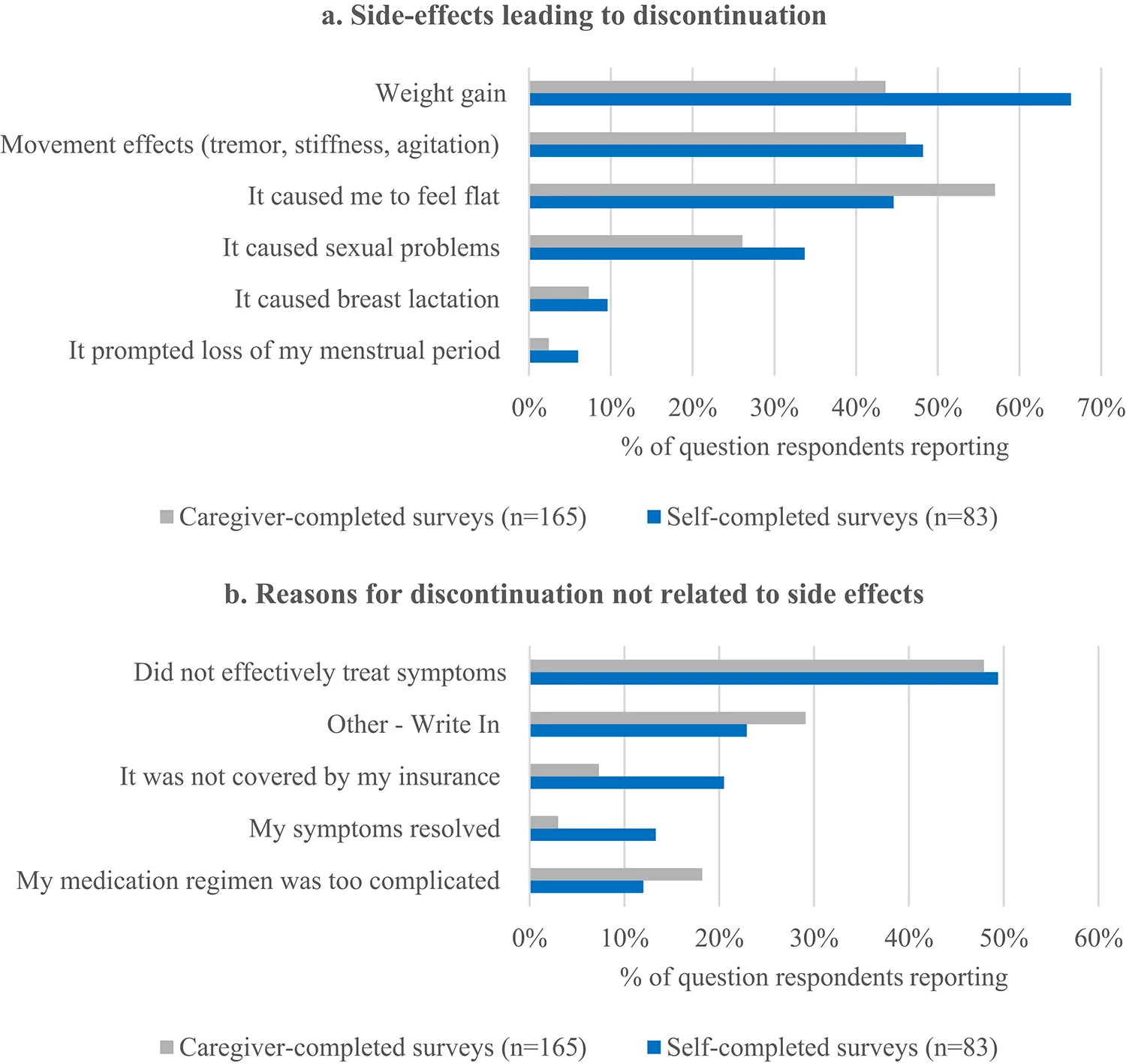
Reasons for discontinuing medications prescribed to treat schizophrenia. Survey questions: Have you ever quit taking a medication prescribed to treat your schizophrenia for any of the following reasons? Check all that apply. *Caregiver Version:* Has the person you care for ever quit taking a medication prescribed to treat their schizophrenia for any of the following reasons? Check all that apply

**Fig. 4:**
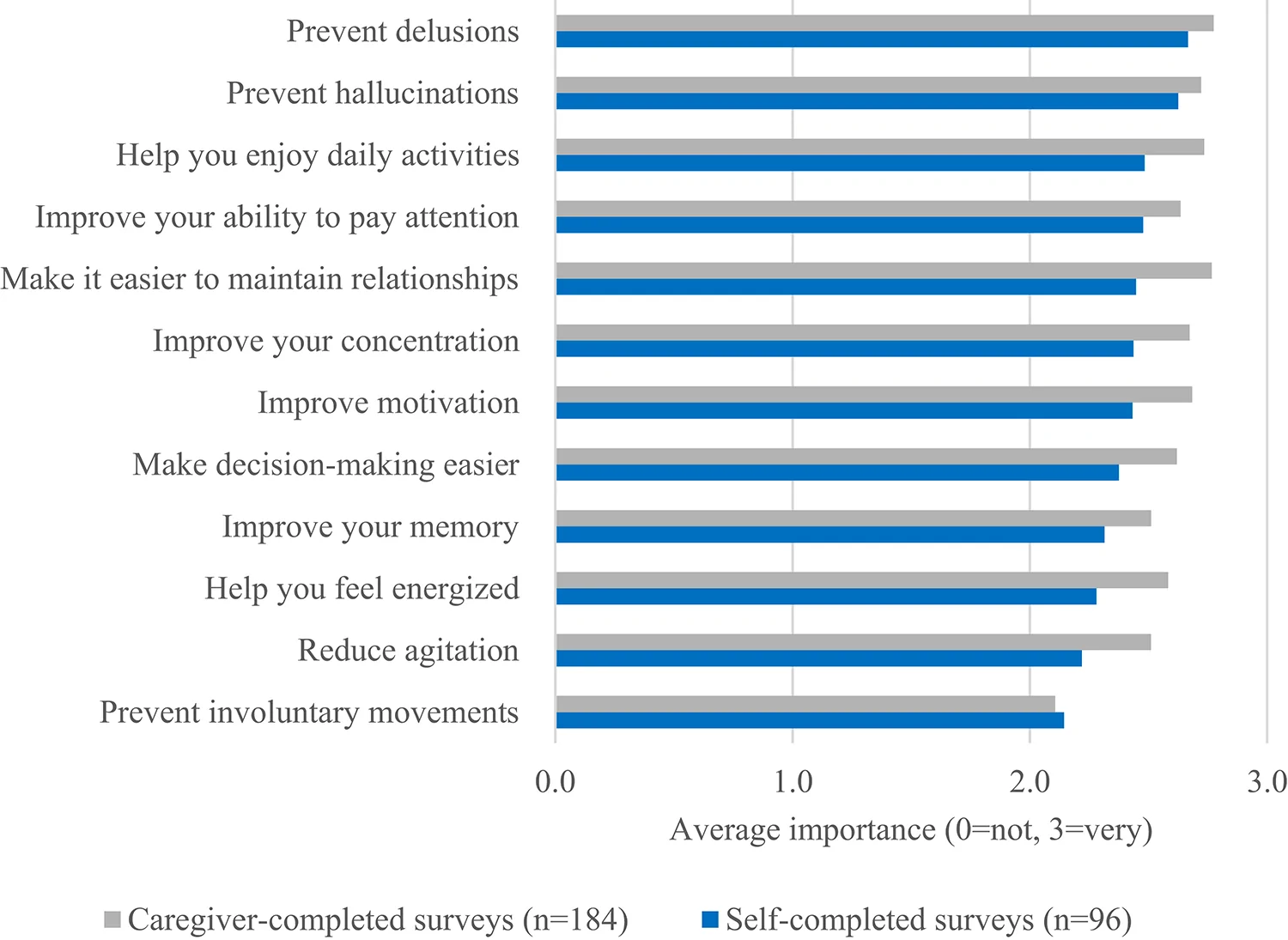
Desired outcomes of future treatments for schizophrenia. Survey questions: When thinking about new treatments for schizophrenia, how important is it that they… *Caregiver Version:* When thinking about new treatments for schizophrenia, how important to them would it be that treatments…

## Discussion

This publication presents insights into daily impacts of schizophrenia, concerns with current medications, and hopes for future schizophrenia treatments. This work builds upon prior research that has demonstrated the sweeping impacts of schizophrenia on daily life while offering expanded insights into experiences with and views on medications for the condition.

These results reinforce the urgent need for treatments that address the profound and multifaceted impacts of schizophrenia. In addition to emphasizing the importance of targeting positive symptoms with future therapies—which has been demonstrated in other work—participants’ responses underscore the disabling effects of negative and cognitive symptoms and their inadequate treatment by current therapies [19, 26–28]. Extant research shows that primary negative symptoms and cognitive symptoms impact about 25% and 80% of those with schizophrenia, respectively, and that medications can cause secondary versions of these symptoms due to their sedative and anticholinergic effects [3]. Medications that specifically target cognitive and negative symptoms, therefore, are greatly needed.

The need for innovation is compounded by the impact of side effects from existing medications. Weight gain, movement effects, and feeling flat were identified as common reasons for quitting medication, and not only diminish the quality of life for those with schizophrenia but also contribute to low medication adherence and poorer outcomes overall. These concerns align with other community-reported treatment experiences and preferences [26, 28, 29]. Because partial adherence and nonadherence are associated with higher rates of relapse, rehospitalization, and suicide attempts, these side effects must be an integral consideration in drug development moving forward [30–32]. Additional research on the barriers to adherence is warranted, as is the opportunity to leverage real world evidence that will refine physician knowledge around best individual treatment choice.

In addition to the new perspectives that it provides, strengths of this study include its sample size, the hybridization of quantitative and qualitative data, and the direct inclusion of lived experience perspectives in data interpretation and manuscript development. This research was commissioned by a schizophrenia advocacy organization, and the authors include a person with schizophrenia and a caregiver. Publication of this research is also important in its own right: while EL-PFDD meeting surveys are becoming increasingly common, their publication in peer-reviewed journals is less so. This manuscript ensures that this work is broadly accessible to researchers, clinicians, and the schizophrenia community writ large, in a durable format that complements the personal narratives shared in the earlier VoP report.

The schizophrenia EL-PFDD meeting—which included preliminary reporting on these survey findings—has informed multiple regulatory decisions. FDA’s Integrated Review for Cobenfy indicates that the meeting report was considered during the review process [33]. Additionally, the meeting highlighted concerns about the clozapine Risk Evaluation and Mitigation Strategy, viewed as overly restrictive and a key barrier to a treatment that many patients credited with restoring their ability to attend school and work and live healthy and productive lives: this meeting feedback helped catalyze further evaluation that ultimately resulted in removal of the REMS program [34].

This study has several limitations. First, the three-month timeline for survey development, aimed to facilitate planning for the EL-PFDD meeting in 2022, precluded thorough vetting or testing of the survey instrument (*e.g.*, cognitive testing). This may have led to varying interpretations of the questions by respondents. Second, the research relies on self-reported data that is subject to recall and social desirability bias. Recall bias is a lesser concern for the questions prioritized within this manuscript, many of which reflect individual viewpoints. However, social desirability bias may have led respondents to provide answers that they considered more socially acceptable than they perceive their true experiences or views to be. Further, caregiver proxy reports may not accurately reflect the views of the people they represent. Selection bias also is a concern. Survey participation may indicate a higher degree of engagement with one’s health and the health of one’s loved ones. Additionally, those with schizophrenia who completed the survey may have less severe or more effectively managed current symptoms than the general schizophrenia population—particularly because participating in this survey required internet access and use, which may indicate higher functioning and greater resource access. These considerations—and the lack of detailed demographic data on the US-based schizophrenia population to compare with the sample as well as the omission of people under 18, who were not eligible to participate, but whose views merit study—limit the generalizability of these findings. All results should be viewed as exploratory and hypothesis-generating.

Finally, because this is an exploratory study and the dataset does not include substantial data that could be used to better characterize the duration of disease or severity of symptoms experienced by people represented within the survey, analyses exploring associations within the data were not attempted. For example, neither stratification nor the use of regression analyses to explore whether treatment preferences differed based upon demographic or disease-related factors were conducted. These topics merit examination in future research.

## Conclusions

As exploratory work that describes community treatment priorities, this research can inform medical product development within schizophrenia moving forward. This publication provides context for drug developers, regulatory agencies, and clinicians who are interested in understanding what matters most to people with schizophrenia. The results may also be useful in examining existing outcome measures (*e.g.*, in assessing content validity) and laying a foundation for future work to develop fit-for-purpose outcome measures. Additionally, this study speaks to the importance of systematic assessment of side effects in schizophrenia clinical trials. While certain side effects are systematically measured as adverse events, many subjective or subtle effects—such as cognitive dulling or emotional blunting—are underreported or inconsistently assessed. The high percentage of respondents who reported discontinuing medication due to side effects underscores the need for standardized tools to capture a broader range of side effects in schizophrenia, both in clinical trials and in real-world use. Finally, the approach, findings, and lessons learned may be beneficial to other disease communities who are interested in PFDD and seeking models for capturing and communicating relevant insights from people with lived experience.

## Supplementary Information

Below is the link to the electronic supplementary material.

Supplementary Material 1.

## Data Availability

Results of our data analysis are provided within the manuscript and supplementary information files.
