# Supplementary material for: Schizophrenia Impacts and Treatment Priorities: Results of An Externally-Led Patient-Focused Drug Development Meeting Survey
Source: Ther Innov Regul Sci. 2026 Jul 23;60(6):1678–86. doi: 10.1007/s43441-026-01001-2 (PMC13722809; doi:10.1007/s43441-026-01001-2)
Supplement: Supplementary file 1 [file 43441_2026_1001_MOESM1_ESM.pdf]

Supplemental Material

Disease Impacts and Treatment Priorities in Schizophrenia: Results of An Externally-Led Patient-Focused Drug Development Meeting Survey

Ilse S. Peterson, Ramaa Chitale, Theresa Frangiosa, Holly B. Krasa, Paulie Benjamin, Anita Fisher, Jessica T. Markowitz, Arun Nagendra*

**Corresponding author*

Contents

A. Tables corresponding to figures and qualitative data in main text of manuscript....................2

Supplemental table 1. Reported impact of schizophrenia symptoms....................2

Supplemental table 2. Effect of schizophrenia on key aspects of daily living....................4

Supplemental table 3: Codebook for analysis of question "What have been the most significant impacts of schizophrenia on your life?"....................5

Supplemental table 4: Frequencies of codes and thematic categorization for open-ended question "what have been the most significant impacts of schizophrenia on your life?"....................7

Supplemental table 5. Reasons for discontinuing medications prescribed to treat schizophrenia....................8

Supplemental table 6. Desired attributes of future treatments for schizophrenia....................9

B. EL-PFDD pre-meeting survey from Voice of the Patient Report....................10

A. Tables corresponding to figures and qualitative data in main text of manuscript

Supplemental table 1. Reported impact of schizophrenia symptoms

Survey Items	Reported impact											
	Self-completed surveys (n=96)						Caregiver proxy surveys (n=186)*					
	# responses	% No impact	% Minimal impact	% Moderate impact	%Significant impact	Weighted average**	# responses	% No impact	% Minimal impact	% Moderate impact	%Significant impact	Weighted average**
**Reported impact of positive and negative symptoms on daily functioning**												
Thoughts about losing control and harming yourself or others	95	32.6	24.2	32.6	10.5	1.2	181	24.9	27.1	24.3	23.8	1.5
Experiences with telepathy, psychic forces, or fortune telling	94	35.1	21.3	24.5	19.1	1.3	181	44.8	14.4	17.7	23.2	1.2
Feeling convinced that you were extremely special or talented	94	27.7	24.5	23.4	24.5	1.4	181	31.5	22.1	18.8	27.6	1.4
Feelings of being watched	94	21.3	12.8	30.9	35.1	1.8	182	13.2	20.9	21.4	44.5	2.0
Seeing, hearing, tasting, smelling, or feeling things that others do not	95	17.9	20.0	21.1	41.1	1.9	184	9.2	12.0	19.0	59.8	2.3
Believing something that others don't believe	95	16.8	20.0	20.0	43.2	1.9	184	5.4	7.6	21.7	65.2	2.5
Loss of interest in daily activities	94	13.8	17.0	31.9	37.2	1.9	185	2.7	9.2	22.7	65.4	2.5
Feeling more afraid that usual	95	14.7	16.8	28.4	40.0	1.9	182	6.6	17.6	34.1	41.8	2.1
Not trusting other people	96	13.5	18.8	27.1	40.6	1.9	185	8.6	15.1	23.8	52.4	2.2
Loss of motivation	95	11.6	16.8	29.5	42.1	2.0	185	3.2	8.6	15.1	73.0	2.6
Loss of interest in social activities	95	11.6	20.0	20.0	48.4	2.1	185	4.9	5.4	24.3	65.4	2.5
Lack of energy	92	13.0	14.1	22.8	50.0	2.1	183	2.7	9.8	32.2	55.2	2.4
**Reported difficulty with items reflective of cognitive functioning (past six months)**												
Speaking so that others can understand me	96	45.8	29.2	17.7	7.3	0.9	184	21.2	29.9	32.6	16.3	1.4
Solving problems	96	33.3	30.2	24.0	12.5	1.2	185	3.8	15.7	40.0	40.5	2.2
Learning new information	95	32.6	25.3	26.3	15.8	1.3	184	5.4	22.8	32.6	39.1	2.1
Controlling emotions	95	29.5	31.6	22.1	16.8	1.3	185	14.1	30.8	27.6	27.6	1.7
Relating to others	96	30.2	28.1	20.8	20.8	1.3	183	4.9	15.3	33.9	45.9	2.2
Making decisions	96	26.0	30.2	26.0	17.7	1.4	184	2.7	15.8	38.0	43.5	2.2
Taking care of yourself	96	32.3	24.0	19.8	24.0	1.4	183	8.7	20.8	35.0	35.5	2.0
Focusing on conversations	96	25.0	32.3	21.9	20.8	1.4	184	3.8	21.2	40.2	34.8	2.1
Expressing emotions	96	24.0	30.2	26.0	19.8	1.4	184	9.2	17.9	38.0	34.8	2.0
Thinking clearly	96	22.9	32.3	22.9	21.9	1.4	184	2.2	18.5	28.8	50.5	2.3
Concentrating on tasks	95	24.2	24.2	30.5	21.1	1.5	185	0.5	11.9	41.1	46.5	2.3
Starting and/or finishing things	96	28.1	17.7	24.0	30.2	1.6	184	3.3	11.4	31.5	53.8	2.4
Remembering things	96	18.8	31.3	24.0	26.0	1.6	185	7.0	21.1	33.0	38.9	2.0
Working	95	24.2	22.1	16.8	36.8	1.7	185	3.2	8.6	13.5	74.6	2.6

Survey questions:

1.Schizophrenia may make it difficult to think, act, or live the way one wants. Have any of the following diminished your ability to function and live the way you would like?
2.Schizophrenia may make it difficult to think, act, or live the way one wants. Have any of the following diminished the ability of the person you care for to function and live the way they would like?

3.In the past six months, have you had difficulty with any of the following?

4.In the past six months, has the person you care for had difficulty with any of the following?

*n=185 for cognitive items

**Weights: Not=0, minimally=1, moderately=2, very=3

Supplemental table 2. Effect of schizophrenia on key aspects of daily living

	Reported change in ability to perform activity of daily life since onset of schizophrenia symptoms															
	Self-completed surveys (n=95)								Caregiver-completed surveys (n=186)							
Aspect of daily life	# responses	% Worsened significantly	% Worsened	% No change	% Improved	% Improved significantly	N/A	Weighted Average	# responses	% Worsened significantly	% Worsened	% No change	% Improved	% Improved significantly	N/A	Weighted Average
Perform at work	95	29.5	25.3	7.4	13.7	8.4	15.8	-0.54	184	52.7	17.4	6.0	4.3	0.5	19.0	-1.2
Maintain relationships	95	26.3	28.4	10.5	20.0	11.6	3.2	-0.38	186	52.7	27.4	7.5	8.1	3.2	1.1	-1.2
Perform in school	94	23.4	16.0	12.8	16.0	5.3	26.6	-0.36	183	45.4	18.0	6.6	3.3	0.0	26.8	-1.1
Manage your responsibilities at home	95	21.1	30.5	18.9	13.7	12.6	3.2	-0.34	185	45.9	33.5	11.4	5.9	1.6	1.6	-1.2
Manage your finances	95	20.0	27.4	15.8	25.3	7.4	4.2	-0.27	186	59.1	20.4	10.2	4.3	0.0	5.9	-1.3
Cope without alcohol or drugs	94	10.6	11.7	21.3	9.6	10.6	36.2	-0.02	185	23.8	16.2	11.9	10.8	9.7	27.6	-0.3

Survey questions:

5.Since you first started experiencing symptoms of schizophrenia, has your ability to do the following changed?

6.Since the person you care for first started experiencing symptoms of schizophrenia, has their ability to do the following changed?

*Weights: Worsened significantly=-2, worsened=-1, no change=0, improved=1, improved significantly=2

Supplemental table 3: Codebook for analysis of question "What have been the most significant impacts of schizophrenia on your life?"

Code	When to apply	When not to apply
**[Category] Symptom**	•When the respondent describes positive, negative, and cognitive symptoms related to schizophrenia. Anosognosia not one of the three core symptoms	•When respondent does not describe positive, negative, or cognitive symptom. When the focus of the response is on impact on life. When the respondent refers to anosognosia
**[Category] Impact on life**	•When the respondent describes any impact on their life because of schizophrenia, managing, or caring for another person with schizophrenia	•When the respondent does not describe impact on their life. Examples might be inability to keep a job, impacts on relationships, use of drugs/alcohol, side effects of medication etc.
**Fear/Anxiety/Stress**	•When the respondent refers to fear, anxiety, worry or tension in their life	•When respondent does not reference feeling fearful, scared<br>•When the respondent does not describe anxiety or worry
**Trust**	•When respondent describes not trusting others, or not trusting the mental health/health care system	•When the respondent does not refer to trusting others or the mental health/ health care system
**Harm to self/others/property**	•When respondent describes attempt at suicide, harming themselves or others<br>•When the respondent describes anger or property damage<br>•When respondent describe anger as behavioral issue	•When respondent does not describe harming themselves or others
**Trauma**	•When the respondent refers to trauma associated with schizophrenia. This might include trauma from hallucinations or in the hospital (treatment team)<br>•For caregivers, the respondent might describe trauma associated with caring for their loved ones<br>•Both caregivers and people with schizophrenia can experience trauma from the illness	•When the respondent does not describe trauma associated with schizophrenia
**Loss of future/delay**	•When respondent describes a loss of some promise in the future (job, career, children, marriage, etc.). Could also describe the delay in future<br>•Apply to temporal situation	•When the respondent does not refer to the loss of future.
**Impacted everything**	•When the respondent uses the term impact everything or affects everything - meaning that schizophrenia is a part of every aspect of their life<br>•Also include general comments about how the illness negatively impacts (example might be words about disabling nature of illness)	•When respondent has specific parts of life that are impacted. For instance, do not apply the code if the respondent is discussing maintaining relationships only or performing at work or school only.

**Social functioning**	•When the respondent describes not having positive relationships with others; social withdrawal - loss of friendship, loss of socialization<br>•When the respondent discusses feeling isolated from others/relationships. This includes feeling isolated from family, friends, society, etc.	•When the respondent does not describe any relationship with others<br>•When the respondent does not discuss feeling isolated from other relationships
**Vocational performance (work and school)**	•When the respondent describes having the ability to perform at work or at school. They may also describe the potential for working, or the potential to complete a degree	•When the respondent does not describe performing at work or school
**Independent living/self-care skills**	•When the respondent describes impact on managing their responsibilities at home like cooking, cleaning, paying bills, etc.<br>•Language might include living independently or what is needed to live independently (example: the need for a caregiver)<br>•When the respondent describes managing the responsibilities of hygiene and self-care	•When the respondent does not refer to common responsibilities in the home
**Cope with alcohol or drugs**	•When respondent describes dealing with symptoms using drugs and alcohol.	•When the respondent does not describe dealing with symptoms using drugs and alcohol
**Side effects from medication**	•When respondent notes side-effects from medication. For instance, weight gain, feeling flat, etc.	•When the respondent does not refer to side-effects from medication
**Homelessness**	•When the respondent describes experiencing homelessness	•When the respondent does not describe experiencing homelessness
**Legal challenges**	•When the respondent describes going to prison, jail, or being incarcerated. Might include mention of being arrested.<br>•This could include the threat of going to prison/could be mental health court	•When the respondent does not mention prison, jail, or being incarcerated
**Self-esteem / self-worth**	•When respondent refers to their self-worth or self-esteem because of the impact on their life	•When the respondent does not refer to their self-esteem or self-worth
**Depression/hopelessness**	•When the respondent describes depression from schizophrenia; when the respondent discusses hopelessness, emptiness, despair	•When the respondent does not describe depression from schizophrenia
**Discrimination and stigma**	•When the respondent describes stigma that they face because of the condition	•When the respondent does not describe stigma
**Physical health/Wellness**	•Sleeping as part of physical health, or mention physical health or generally not feeling well<br>•Could also be related to weight gain	•When the respondent is not describing physical health or anything related to wellness
**Positive**	•When the respondent notes a positive change from treatment of schizophrenia	•When the respondent does not note a positive change from schizophrenia

Supplemental table 4: Frequencies of codes and thematic categorization for open-ended question "what have been the most significant impacts of schizophrenia on your life?"

**Theme**	**Code**	Frequency (%)	
		**Self-completed surveys (n=71)**	**Caregiver proxy surveys (n=176)**
Lost opportunities	Vocational performance (work and school)	42.3	45.5
	Independent living/self-care skills	14.1	27.8
	Physical health/Wellness	12.7	23.3
	Loss of future/delay	11.3	14.8
Safety concerns	Harm to self/others/property	8.5	10.2
	Side effects from medication	5.6	6.8
	Homelessness	2.8	6.8
	Legal challenges	1.4	5.1
	Cope with alcohol or drugs	1.4	5.7
	Discrimination and stigma	1.4	0.6
Impacts on social functioning	Social functioning	42.3	58.0
	Impacted everything	12.7	13.6
	Fear/Anxiety/Stress	11.3	5.7
	Trauma	4.2	0.6
	Trust	2.8	3.4
	Depression/hopelessness	2.8	2.3
	Self-esteem / self-worth	1.4	2.8
Positive impacts	Positive	2.8	0.6

Supplemental table 5. Reasons for discontinuing medications prescribed to treat schizophrenia

Reason	% of respondents selecting item	
	Self-completed surveys (n=83)	Caregiver proxy surveys (n=165)
Undesirable side effects		
It prompted loss of my menstrual period	6.0	2.4
It caused breast lactation	9.6	7.3
It caused sexual problems	33.7	26.1
It caused me to feel flat	44.6	57.0
Movement effects (tremor, stiffness, agitation)	48.2	46.1
Weight gain	66.3	43.6
My medication regimen was too complicated	12.0	18.2
My symptoms resolved	13.3	3.0
It was not covered by my insurance	20.5	7.3
Other - Write In	22.9	29.1
Did not effectively treat symptoms	49.4	47.9

Survey questions:
15.Have you ever quit taking a medication prescribed to treat your schizophrenia for any of the following reasons? Check all that apply.
16.Has the person you care for ever quit taking a medication prescribed to treat their schizophrenia for any of the following reasons? Check all that apply.

Supplemental table 6. Desired attributes of future treatments for schizophrenia

Attribute	Reported Importance of Attributes											
	Self-completed surveys (n=96)						Caregiver proxy surveys (n=184)					
	n	Not %	Minimally %	Moderately %	Very %	Weighted average*	n	Not %	Minimally %	Moderately %	Very %	Weighted average*
Prevent involuntary movements	96	10.4	15.6	22.9	51.0	2.1	180	13.3	14.4	20.6	51.7	2.1
Reduce agitation	96	8.3	14.6	24.0	53.1	2.2	182	2.7	10.4	19.8	67.0	2.5
Help you feel energized	96	6.3	12.5	28.1	53.1	2.3	181	1.7	6.6	23.8	68.0	2.6
Improve your memory	95	6.3	14.7	20.0	58.9	2.3	184	3.3	4.3	30.4	62.0	2.5
Make decision-making easier	96	4.2	13.5	22.9	59.4	2.4	183	1.6	3.8	25.7	68.9	2.6
Improve motivation	95	6.3	8.4	21.1	64.2	2.4	184	2.2	3.8	17.4	76.6	2.7
Improve your concentration	96	4.2	11.5	20.8	63.5	2.4	184	1.1	3.3	22.8	72.8	2.7
Make it easier to maintain relationships	96	4.2	10.4	21.9	63.5	2.4	184	1.1	2.7	14.7	81.5	2.8
Improve your ability to pay attention	94	2.1	11.7	22.3	63.8	2.5	184	1.1	6.0	21.2	71.7	2.6
Help you enjoy daily activities	95	2.1	11.6	22.1	64.2	2.5	184	1.6	3.3	15.2	79.9	2.7
Prevent hallucinations	96	5.2	2.1	17.7	75.0	2.6	181	4.4	3.3	7.7	84.5	2.7
Prevent delusions	96	2.1	5.2	16.7	76.0	2.7	183	3.8	1.1	8.7	86.3	2.8

9.When thinking about new treatments for schizophrenia, how important is it that they...

10.When thinking about new treatments for schizophrenia, how important to them would it be that treatments...

*Weights: Not=0, minimally=1, moderately=2, very=3

B. EL-PFDD pre-meeting survey from Voice of the Patient Report

Pre-Meeting Survey: Schizophrenia Externally Led Patient-Focused Drug Development Meeting
Extracted from the Voice of the Patient Report

What is the purpose of this survey?

The purpose of this survey is to gain greater understanding about what it is like to live with schizophrenia, and what individuals with schizophrenia want from drug treatments. This survey was commissioned by the Schizophrenia & Psychosis Action Alliance (S&PAA) in partnership with the American Foundation for Suicide Prevention, Mental Health America, the National Alliance on Mental Illness, and the National Council for Mental Wellbeing. We are hoping for responses from people living with schizophrenia in the United States, along with their caregivers. This research will help patient organizations, therapy developers, and the U.S. Food and Drug Administration (FDA) better understand what matters when it comes to symptoms, impacts on daily life, and development of new therapies. Survey results will inform planning for a **community-led meeting with the FDA in late 2022**. Summary results will also be shared in a public report after the meeting.

Who can participate?

This survey is for:

- Adults with schizophrenia who received their diagnosis at least six months ago
- Caregivers of adults with schizophrenia who are not able to complete this survey on their own behalf

It is not intended for professional, paid caregivers.

Respondents must live in the United States and have reached the age of majority, which is 19 years old in Alabama and Nebraska, 21 in Mississippi and Puerto Rico, and 18 in all other states.

What does the survey ask?

This survey asks for information about when and how you or the person you provide care for received a diagnosis of schizophrenia and how the disease has affected your life or theirs. It includes questions about symptoms and their impact on things like relationships, work, school, and overall health. It also asks about experiences with drug treatments and other approaches to managing the disease, what you'd like to see from future drug treatments, and views about participating in research. Many questions are multiple choice. You will also have the option to note any other things that you would like us to know.

What do I need to know before continuing?

Participation is optional. If you begin the survey, you can stop at any time. Before beginning, you will be asked to complete an informed consent form. That form explains more about this research and how it will be used, as well as how your information will be protected. If you have any questions about this study, you can contact the research team via **this form**. You may also contact the Advarra Institutional Review Board (IRB) with questions about research participants' rights or read more **here**.

Action: Section Navigator

Are you an individual with schizophrenia or the caregiver of an individual with schizophrenia? Please select "Answer" next to whichever applies.

If you care for more than one person with schizophrenia, please complete separate surveys for each individual. You can do this by completing a survey for one individual and then returning to the survey homepage to complete the next.

Action: JavaScript
New JavaScript

Consent to Participate

Page description:

This form has important information about why this survey is being done, what participation involves, and how the information survey respondents provide will be used.

Page exit logic: Skip / Disqualify Logic

IF: (#1 Question "Please check each of the boxes below to indicate your understanding of these terms and consent to participate.

" is not exactly equal to ("I have read this consent form, or it has been read to me. I understand the information in this form, have had an opportunity to ask questions, and have had my questions answered. If I have additional questions, I understand that I can contact the research team. I agree to participate in this study and agree that the information I provide can be used as described.","I have reached the age of majority (19 in Alabama and Nebraska, 21 in Mississippi and Puerto Rico, and 18 in all other states).","I live in the United States.") OR #3 Question "Please enter your age in years" is less than "18") THEN: Disqualify and display:

Thank you for your interest in this survey. This survey is only for individuals who have reached the age of majority, and respondents must indicate agreement with all terms listed on the prior page. If you are an individual with schizophrenia who is younger than the age of majority, we encourage you to ask your parent or legal guardian of majority age to complete the survey on your behalf. Please contact the research team if you have any questions.

What are the goals of this study?

You are being asked to participate in a research study about your experience living with schizophrenia. The study's goals are to:

- Provide insight into the frequency, severity, and significance of symptoms experienced by people with the schizophrenia;
- Describe how schizophrenia symptoms impact daily life;
- Describe approaches that people with schizophrenia use to treat and manage the disease and its symptoms;
- Provide perspective on why people do or do not use medications prescribed for schizophrenia; and
- Identify desired outcomes of schizophrenia medications that might be developed in the future.

This survey was commissioned by the Schizophrenia and Psychosis Action Alliance (S&PAA) in partnership with the American Foundation for Suicide Prevention, Mental Health America, the National Alliance on Mental Illness, and the National Council for Mental Wellbeing. It is being conducted by Faegre Drinker Consulting (Faegre Drinker).

What will I do if I choose to participate?

You will be asked to complete a survey about your experience with schizophrenia and what you would like from therapies for schizophrenia. The survey should take about 15 minutes.

What are my rights as a research participant?

Participation in this study is voluntary. If you begin the survey, you can stop at any time. You do not have to answer any question you do not want to answer. You will not be penalized if you decide to quit the survey.

How will you use the information that you collect?

This research will help patient organizations, therapy developers, and the U.S. Food and Drug Administration (FDA) better understand what matters when it comes to symptoms, impacts on daily life, and development of new therapies. Survey results will inform planning for a community-led meeting with the FDA in late 2022. Summary results will also be shared in a public report after the meeting. Results of this study also may be used in subsequent publications and presentations. If results of this study are published or presented, individual names and other personally identifiable information will be removed. We may share the data we collect from you with other researchers but will not share any of your personal identifiable information.

What benefits will I get from participating?

This study is designed to learn more about the experiences and views of those living with and caring for people with schizophrenia. Study results may be used in the future to help individuals with schizophrenia, as well as their caregivers and families, and inform U.S. Food and Drug Administration decisions about therapies for schizophrenia. You are not likely to have any direct benefits from participating.

What are the risks or discomforts associated with participation?

This survey does not involve any significant physical risks. As with all research, there is a chance that confidentiality of the information we collect about you could be breached. We take steps to minimize this risk, as described below.

Because the survey asks about experiences with schizophrenia, it is possible that some questions could be upsetting. If you find yourself in distress, help is available:

- The NAMI HelpLine—800-950-6264 or info@nami.org—can offer sympathy and support and provide information about resources in your community. To find a warmline in your area, dial 211, or go to www.211.info, for information on local social services.
- The American Foundation for Suicide Prevention provides information about suicide and preventing suicide as well as support for those affected by suicide. Reach the Crisis Text Line at crisistextline.org, call 1-800-273-8255, or text TALK to 741741 to connect with a trained crisis counselor for free, 24/7.
- The National Suicide Prevention Lifeline provides 24/7, free and confidential support for people in distress, prevention and crisis resources for you or your loved ones, and best practices for professionals. Find more information about the Lifeline at suicidepreventionlifeline.org or dial 800-273-8255.

How will the information I provide be protected?

This survey is hosted on the Alchemer platform, which encrypts data and can only be accessed through secure HTTPS. Data will be analyzed by a team of individuals from Faegre Drinker and S&PAA. All personal information collected via the survey will remain confidential to this team, and all information shared in papers or reports on this research will be deidentified. Additionally, any data stored for the long term or used in comparison to other data in future research will be de-linked from identity data. Neither S&PAA nor Faegre Drinker will sell your data. By submitting personal information through this survey or to a Faegre Drinker or S&PAA contact, you agree that Faegre Drinker and S&PAA may contact you with questions related to the survey.

Who can I contact if I have questions or concerns about this study?

If you have any questions about this study, you can contact the research team via this form. You may also contact Advarra Institutional Review Board (IRB) with questions about research participants' rights or read more here.

VALIDATION

Min. answers = 3 (if answered)

1. Please check each of the boxes below to indicate your understanding of these terms and consent to participate.

- ▯ I have read this consent form, or it has been read to me. I understand the information in this form, have had an opportunity to ask questions, and have had my questions answered. If I have additional questions, I understand that I can contact the research team. I agree to participate in this study and agree that the information I provide can be used as described.
- ▯ I have reached the age of majority (19 in Alabama and Nebraska, 21 in Mississippi and Puerto Rico, and 18 in all other states).
- ▯ I live in the United States.

2. Please sign this form by completing each of the fields below: *

First Name __________

Last Name __________

VALIDATION

Must be numeric Whole numbers only Positive numbers only

LOGIC

Hidden unless: QUESTION NOT FOUND! is one of the following answers [NO OPTIONS SET]

3. Please enter your age in years * __________

Part 1. Initial Symptoms and Diagnosis

Page description:

As you continue with this survey, you will be asked questions about initial schizophrenia symptoms and diagnosis, daily life, research, and disease management and treatment. Try to answer as accurately as you can. If you don't know the answer to a question, it is OK to leave it blank.

VALIDATION

Must be numeric Whole numbers only Positive numbers only

4. How many years old were you when...

You first experienced symptoms of schizophrenia?	
You were officially diagnosed with schizophrenia?	

5. Before receiving a diagnosis of schizophrenia, did you experience any of the following health-related events or circumstances? Check all that apply.

- ☐ Started eating less regularly
- ☐ Had trouble sleeping
- ☐ Increased use of alcohol or drugs
- ☐ Received treatment for substance use disorder
- ☐ Had thoughts of self-harm
- ☐ Attempted suicide
- ☐ Was hospitalized for psychiatric reasons

6. Before receiving a diagnosis of schizophrenia, did you experience any of the following life events or circumstances? Check all that apply.

- ☐ Struggled with relationships
- ☐ Had difficulty doing my job
- ☐ Lost my job
- ☐ Had difficulty in school
- ☐ Was expelled from school
- ☐ Was the victim of a crime or abuse
- ☐ Experienced homelessness
- ☐ Was arrested because of behaviors related to schizophrenia
- ☐ Was incarcerated in prison or jail
- ☐ Needed legal services not related to being arrested (for example, help with legal guardianship, disability rights, other civil matters)
- ☐ Needed increased special education or employment support services

7. Around the time you received a diagnosis of schizophrenia, did you experience any of the following? Check all that apply.

- Thoughts or fears related to cleanliness and contamination
- Difficulty tolerating uncertainty or disorganization
- Thoughts about harming yourself or others
- Repetitive behaviors such as frequent washing, cleaning, counting, or demanding reassurance
- Loss of motivation
- Loss of interest in daily activities
- Loss of interest in social activities
- Lack of energy
- Seeing, hearing, tasting, smelling, or feeling things that others did not
- Feeling convinced that you were extremely special or talented
- Experiences with telepathy, psychic forces, or fortune telling
- Feeling more afraid than usual
- Feeling you were being watched
- Not trusting other people
- Believing things others didn't believe

8. Around the time you received a diagnosis of schizophrenia, did you have difficulty with any of the following? Check all that apply.

- Expressing emotions
- Controlling emotions
- Speaking clearly so that others can understand me
- Thinking clearly
- Relating to others
- Focusing on conversations
- Concentrating on tasks
- Remembering things
- Learning new information
- Solving problems
- Making decisions
- Taking care of yourself
- Working
- Starting things and/or finishing things

9. Have any of your relatives been diagnosed with schizophrenia or a related condition? If so, please check the box(es) for anyone who has.

- ▯ No, not to my knowledge
- ▯ Brother or sister
- ▯ Cousin
- ▯ Parent
- ▯ Aunt or uncle
- ▯ Grandparent
- ▯ Niece or Nephew
- ▯ Other – Please Describe: ____________________

LOGIC

Show/hide trigger exists.

10. Before you were diagnosed with schizophrenia, did you receive any other diagnoses to explain the symptoms that you were experiencing?

- ▯ Yes
- ▯ No

LOGIC

Hidden unless: #10 Question "Before you were diagnosed with schizophrenia, did you receive any other diagnoses to explain the symptoms that you were experiencing?" is one of the following answers ("Yes")

11. Did you receive any of the following diagnoses? Check all that apply.

- ▯ Anxiety (e.g., generalized anxiety disorder, social anxiety disorder, phobias)
- ▯ Attention deficit hyperactivity disorder (ADHD)
- ▯ Autism Spectrum Disorder
- ▯ Bipolar disorder
- ▯ Major depressive disorder (MDD)
- ▯ Post-traumatic stress disorder (PTSD)
- ▯ Borderline personality disorder
- ▯ Traumatic brain injury (TBI)
- ▯ Delusional disorder
- ▯ Obsessive compulsive disorder (OCD)
- ▯ Schizoaffective disorder
- ▯ Schizophreniform disorder
- ▯ Substance use disorder
- ▯ Other Diagnosis – Please Specify: ____________________

12. In addition to schizophrenia, do you currently have an official diagnosis of any of the following? Check all that apply.

- ☐ Anxiety, including generalized anxiety disorder, social anxiety disorder, and phobias
- ☐ Attention deficit hyperactivity disorder (ADHD)
- ☐ Autism Spectrum Disorder
- ☐ Bipolar disorder
- ☐ Borderline personality disorder
- ☐ Delusional disorder
- ☐ Major depressive disorder (MDD)
- ☐ Obsessive compulsive disorder
- ☐ Post-traumatic stress disorder (PTSD)
- ☐ Schizoaffective disorder
- ☐ Schizophreniform disorder
- ☐ Substance use disorder
- ☐ Traumatic brain injury (TBI)
- ☐ Other Diagnosis – Please Specify:

Part 2. Living with Schizophrenia: Symptoms

13. Schizophrenia may make it difficult to think, act, or live the way one wants. Have any of the following diminished your ability to function and live the way you would like?

	No Impact	Minimal Impact	Moderate Impact	Significant Impact
Thoughts or fears related to cleanliness and contamination	❍	❍	❍	❍
Difficulty tolerating uncertainty or disorganization	❍	❍	❍	❍
Thoughts about losing control and harming yourself or others	❍	❍	❍	❍
Repetitive behaviors such as frequent washing, cleaning, counting, or demanding reassurance	❍	❍	❍	❍
Loss of motivation	❍	❍	❍	❍
Loss of interest in daily activities	❍	❍	❍	❍
Loss of interest in social activities	❍	❍	❍	❍
Lack of energy	❍	❍	❍	❍
Seeing, hearing, tasting, smelling, or feeling things that others do not	❍	❍	❍	❍
Feeling convinced that you were extremely special or talented	❍	❍	❍	❍
Experiences with telepathy, psychic forces, or fortune telling	❍	❍	❍	❍
Feeling more afraid than usual	❍	❍	❍	❍
Feelings of being watched	❍	❍	❍	❍
Not trusting other people	❍	❍	❍	❍
Believing something that others don't believe	❍	❍	❍	❍

14. In the past six months, have you had difficulty with any of the following?

	No	Minimal Difficulty	Moderate Difficulty	Significant Difficulty
Expressing emotions	❍	❍	❍	❍
Controlling emotions	❍	❍	❍	❍
Speaking so that others can understand me	❍	❍	❍	❍
Thinking clearly	❍	❍	❍	❍
Relating to others	❍	❍	❍	❍
Focusing on conversations	❍	❍	❍	❍
Concentrating on tasks	❍	❍	❍	❍
Remembering things	❍	❍	❍	❍
Learning new information	❍	❍	❍	❍
Solving problems	❍	❍	❍	❍
Making decisions	❍	❍	❍	❍
Taking care of yourself	❍	❍	❍	❍
Working	❍	❍	❍	❍
Starting and/or finishing things	❍	❍	❍	❍

15. In the past 12 months, have you done or experienced any of the following? Check all that apply. ***Please remember that all individual responses will remain confidential, and honest answers will help ensure that we are accurately representing the schizophrenia community.***

- Received treatment for substance use disorder
- Been the victim of a crime or abuse
- Experienced homelessness
- Had thoughts of self-harm
- Attempted suicide
- Been hospitalized for psychiatric reasons
- Been arrested
- Been incarcerated in prison or jail
- Needed other legal services (including but not limited to legal guardianship, disability rights, other civil matters)
- Needed special education or employment support services beyond my usual needs
- Overdosed on drugs
- Other – Write In

16. How well do the below statements describe you and your experience today?

	Agree very much	Agree	Unsure	Disagree	Disagree very much
Some of the symptoms were made up by my mind	❍	❍	❍	❍	❍
I do not need medication	❍	❍	❍	❍	❍
The doctor is right in prescribing medication for me	❍	❍	❍	❍	❍
If someone told me that I had a mental illness they would be right	❍	❍	❍	❍	❍
My stay in the hospital was necessary	❍	❍	❍	❍	❍
None of the unusual things I experienced are due to an illness	❍	❍	❍	❍	❍
I do not need to be seen by a doctor or psychiatrist	❍	❍	❍	❍	❍
I am mentally well	❍	❍	❍	❍	❍

VALIDATION

Must be numeric

17. From the time you received your schizophrenia diagnosis to today, about how many times have you experienced a setback or "recurrence" in your symptoms?

18. How severe would you say your schizophrenia symptoms have been…

	No symptoms	Mild	Moderate	Severe
During the past week	❍	❍	❍	❍
During the past 30 days	❍	❍	❍	❍

19. How much do your schizophrenia symptoms negatively impact your life…

	No Impact	Minimal Impact	Moderate Impact	Significant Impact
On the best days	❍	❍	❍	❍
On the worst days	❍	❍	❍	❍

VALIDATION

Max character count = 100

20. What have been the most significant impacts of schizophrenia on your life?

Part 3. Living with Schizophrenia: Daily Life

21. Which of these best describes your current living situation?

- Living independently, alone
- A household with relatives or friends
- Supervised group housing
- Partially supervised group housing
- Residential substance use program (e.g., sober house, halfway house)
- Supportive housing
- Long-term care facility, such as a nursing home, senior housing, or a skilled nursing facility
- Correctional facility (e.g., prison, jail)
- Shelter
- Currently without housing
- I don't know

22. Have you had any other living situations within the past 12 months? Check all that apply.

- Living independently, alone
- A household with relatives or friends
- Supervised group housing
- Partially supervised group housing
- Residential substance use program (e.g., sober house, halfway house)
- Supportive housing
- Long-term care facility, such as a nursing home, senior housing, or a skilled nursing facility
- Correctional facility (e.g., prison, jail)
- Shelter
- Currently without housing
- I don't know

23. Are you currently employed, or working for pay?

- Yes, full-time
- Yes, part-time
- Actively looking for work but not currently employed
- Not currently employed
- Retired
- Prefer not to say

24. Are you currently in school?

- Yes, full-time
- Yes, part-time
- No

25. Since you first started experiencing symptoms of schizophrenia, has your ability to do the following changed?

	Worsened significantly	Worsened	No change	Improved	Improved significantly	Not applicable
Maintain relationships	❍	❍	❍	❍	❍	❍
Perform at work	❍	❍	❍	❍	❍	❍
Perform in school	❍	❍	❍	❍	❍	❍
Manage your responsibilities at home	❍	❍	❍	❍	❍	❍
Manage your finances	❍	❍	❍	❍	❍	❍
Cope without alcohol or drugs	❍	❍	❍	❍	❍	❍

26. Have you made any of the following changes in your career or education because of schizophrenia symptoms? Check all that apply.

- Cut back on hours at work
- Took a leave of absence from a job
- Took a leave of absence from school
- Switched to a job with fewer or more predictable hours
- Switched to a less challenging job
- Switched to a job that I was overqualified for
- Passed up a promotion or desired job change
- Postponed or cancelled future education plans
- Left the workforce altogether
- Took early retirement
- Other – Write In

27. Have you done any of the following in your personal life because of schizophrenia symptoms? Check all that apply.

- Avoided important social gatherings
- Stopped pursuing hobbies
- Avoided taking vacation
- Ended a romantic relationship
- None of the above
- Other – Write In

28. Have you ever used or taken part in any of the following forms of support? Check all that apply.

- ▯ Financial support from family or friends
- ▯ Financial assistance from the government
- ▯ Disability insurance
- ▯ Supported employment
- ▯ Vocational rehabilitation other than supported employment
- ▯ Clubhouse program
- ▯ Respite care
- ▯ Other - Write In

LOGIC

Show/hide trigger exists.

29. Have you ever had a psychiatric advance directive (PAD) in place? This could include a conservatorship/guardianship, medical or mental health power of attorney, financial power of attorney, or court-mandated treatment.

- ▯ Yes
- ▯ No
- ▯ I don't know
- ▯ None of the above
- ▯ Other - Write In

LOGIC

Hidden unless: #29 Question "Have you ever had a psychiatric advance directive (PAD) in place? This could include a conservatorship/guardianship, medical or mental health power of attorney, financial power of attorney, or court-mandated treatment." is one of the following answers ("Yes")

30. Have you ever been under court-mandated treatment or conservatorship?

- ▯ Yes
- ▯ No
- ▯ I don't know

Part 4. Treating and Managing Schizophrenia

31. When thinking about new treatments for schizophrenia, how important is it that they...

	Not important	Minimally important	Moderately important	Very important
Prevent delusions	❍	❍	❍	❍
Prevent hallucinations	❍	❍	❍	❍
Improve motivation	❍	❍	❍	❍
Help you feel energized	❍	❍	❍	❍
Help you enjoy daily activities	❍	❍	❍	❍
Make it easier to maintain relationships	❍	❍	❍	❍
Make decision-making easier	❍	❍	❍	❍
Improve your ability to pay attention	❍	❍	❍	❍
Improve your concentration	❍	❍	❍	❍
Prevent involuntary movements	❍	❍	❍	❍
Reduce agitation	❍	❍	❍	❍
Enter another option	❍	❍	❍	❍

32. About how many hours per week do you spend actively managing your schizophrenia? This can include time spent attending medical or therapy appointments, traveling to and from appointments, and spending time on your own dealing with or trying to improve symptoms.

- ▯ 0-5 hours
- ▯ 5-10 hours
- ▯ 10-15 hours
- ▯ 15-20 hours
- ▯ More than 20 hours

33. What types of non-drug treatment or support have you received? Are you receiving these currently?

	Not important	Minimally important
Inpatient psychiatric treatment	❍	❍
Substance use disorder treatment	❍	❍
Outpatient psychiatric or psychological treatment (therapy, counsel-ing, or support groups)	❍	❍
Housing support services	❍	❍
Employment or education support services	❍	❍
None of the above	❍	❍

LOGIC
Show/hide trigger exists.

34. Are you currently taking any medications to manage your schizophrenia? Check all that apply.

- ▯ Oral antipsychotic medication
- ▯ Long-acting injectable antipsychotic medication
- ▯ Other psychiatric medication (such as antidepressants or anxiolytics)
- ▯ Other - Write In

LOGIC
Hidden unless: #34 Question "Are you currently taking any medications to manage your schizophrenia? Check all that apply." is one of the following answers ("Oral antipsychotic medication","Long-acting injectable antipsychotic medication","Other psychiatric medication (such as antidepressants or anxiolytics)")

35. Do you take your medication(s) as prescribed by your doctor?

- ▯ Yes
- ▯ No
- ▯ I don't know

VALIDATION
Must be numeric

36. How many different schizophrenia medications have you taken since you were diagnosed with schizophrenia?

37. Have you ever switched from one medication to another for the reasons below? Check all that apply.

- ▯ A medication that I was taking did not effectively treat my symptoms
- ▯ My doctor recommended taking a different medication
- ▯ A medication that I was taking had undesirable side effects
- ▯ My insurance coverage for the new medication was better
- ▯ My insurance stopped covering the medication I was taking
- ▯ Other - Write In

LOGIC

38. Have you ever quit taking a medication prescribed to treat your schizophrenia for any of the following reasons? Check all that apply.

- ☐ My medication regimen was too complicated
- ☐ It did not treat my symptoms
- ☐ It caused me to feel flat
- ☐ It caused me to gain weight
- ☐ It caused movement effects (tremor, stiffness, agitation)
- ☐ It caused sexual problems
- ☐ It prompted loss of my menstrual period
- ☐ It caused breast lactation
- ☐ It was not covered by my insurance
- ☐ My symptoms resolved
- ☐ Other – Write In

Part 5. Research Studies

39. Have you participated in any of the following types of research studies? Check all that apply. If you don't know, select "I don't know."

- Natural history study for schizophrenia – These studies seek to understand how diseases progress or change over time, without treatment.
- Other observational study for schizophrenia – These include other studies that assess health outcomes but do not involve specific interventions, such as changes in diet, exercise, or use of medications or devices.
- Interventional study for schizophrenia that tested the effects of something other than a drug – Examples of interventional studies that are not for investigational drugs include studies for which participants might change their diets, exercise habits, or other activities.
- I don't know

LOGIC
Show/hide trigger exists.

40. Have you ever participated in a clinical trial for an investigational drug for schizophrenia? These studies test the safety and efficacy of medications to determine whether they should be approved for use outside of research studies.

- Yes
- No
- I don't know.

LOGIC
Hidden unless: #40 Question "Have you ever participated in a clinical trial for an investigational drug for schizophrenia? These studies test the safety and efficacy of medications to determine whether they should be approved for use outside of research studies." is one of the following answers ("Yes")

41. How much motivation, if any, did the following provide to participate in the trial?

	Not at all	Minimal Motivation	Moderate Motivation	Significant Motivation
Desire for clinical benefit and improved quality of life	❍	❍	❍	❍
Access to investigational drug	❍	❍	❍	❍
Access to medical care	❍	❍	❍	❍
Access to medical experts	❍	❍	❍	❍
Positive reputation of the study team	❍	❍	❍	❍
Positive interactions with the study team	❍	❍	❍	❍
Opportunity to help others with schizophrenia	❍	❍	❍	❍
Opportunity to contribute to science	❍	❍	❍	❍
Support from their doctor	❍	❍	❍	❍
Suggested by another person whose family is impacted by the disease	❍	❍	❍	❍
Suggested by a patient advocacy organization	❍	❍	❍	❍

LOGIC

Hidden unless: #40 Question "Have you ever participated in a clinical trial for an investigational drug for schizophrenia? These studies test the safety and efficacy of medications to determine whether they should be approved for use outside of research studies." is one of the following answers ("Yes")

42) Did any of the following cause concern about participation in the trial? Check all that apply.

- ☐ Concern about not being accepted into the trial
- ☐ Uncertainty about the safety of the study drug
- ☐ Uncertainty about whether potential benefits would justify potential risks
- ☐ Fear of receiving placebo
- ☐ Fear of physical and/or mental pain that could accompany required tests
- ☐ Uncertainty about whether the study requirements would allow me to manage their schizophrenia in the way that I'd like
- ☐ Time required
- ☐ Travel required
- ☐ Long-term commitment required
- ☐ Complexity of the study logistics
- ☐ Concern about loss of control
- ☐ Level of compensation for participation
- ☐ Other - Write In:

LOGIC
Show/hide trigger exists. Hidden unless: #40 Question "Have you ever participated in a clinical trial for an investigational drug for schizophrenia? These studies test the safety and efficacy of medications to determine whether they should be approved for use outside of research studies." is one of the following answers ("Yes")

43) Would you consider joining a clinical trial again?

- Yes
- No
- I don't know

LOGIC
Logic: Hidden unless: #43 Question "Would you consider joining a clinical trial again?" is one of the following answers ("No")

44) Why not?

LOGIC
Hidden unless: #40 Question "Have you ever participated in a clinical trial for an investigational drug for schizophrenia? These studies test the safety and efficacy of medications to determine whether they should be approved for use outside of research studies." is one of the following answers ("No")

45) Before taking this survey, were you aware that there are research studies on new medications that haven't yet been proven effective but might be?

- Yes
- No
- I don't know

LOGIC
Hidden unless: #40 Question "Have you ever participated in a clinical trial for an investigational drug for schizophrenia? These studies test the safety and efficacy of medications to determine whether they should be approved for use outside of research studies." is one of the following answers ("No")

46) Have you ever tried to participate in a clinical trial but have not been eligible?

- Yes
- No
- I don't know

LOGIC

Hidden unless: #40 Question "Have you ever participated in a clinical trial for an investigational drug for schizophrenia? These studies test the safety and efficacy of medications to determine whether they should be approved for use outside of research studies." is one of the following answers ("No")

47) Would you consider joining a clinical trial in the future?

- Yes
- No
- I don't know

LOGIC

Hidden unless: #40 Question "Have you ever participated in a clinical trial for an investigational drug for schizophrenia? These studies test the safety and efficacy of medications to determine whether they should be approved for use outside of research studies." is one of the following answers ("No","I don't know")

48) If you were given the chance to participate in a clinical trial, how much motivation do you think the following would provide to participate?

	Not at all	Minimal Motivation	Moderate Motivation	Significant Motivation
Desire for clinical benefit and improved quality of life	❍	❍	❍	❍
Access to investigational drug	❍	❍	❍	❍
Access to medical care	❍	❍	❍	❍
Access to medical experts	❍	❍	❍	❍
Positive reputation of the study team	❍	❍	❍	❍
Positive interactions with the study team	❍	❍	❍	❍
Opportunity to help others with schizophrenia	❍	❍	❍	❍
Opportunity to contribute to science	❍	❍	❍	❍
Support from their doctor	❍	❍	❍	❍
Suggested by another person whose family is impacted by the disease	❍	❍	❍	❍
Suggested by a patient advocacy organization	❍	❍	❍	❍

LOGIC

Hidden unless: #40 Question "Have you ever participated in a clinical trial for an investigational drug for schizophrenia? These studies test the safety and efficacy of medications to determine whether they should be approved for use outside of research studies." is one of the following answers ("No","I don't know")

49) If you were given the chance to participate in a clinical trial in the future, how much concern do you think the following might cause you?

	No Concern	Minimal Concern	Moderate Concern	Significant Concern
Concern about not being accepted into the trial	❍	❍	❍	❍
Uncertainty about the safety of the study drug	❍	❍	❍	❍
Uncertainty about whether potential benefits would justify potential risks	❍	❍	❍	❍
Fear of receiving placebo	❍	❍	❍	❍
Fear of physical and/or mental pain that could accompany required tests	❍	❍	❍	❍
Uncertainty about whether the study requirements would allow me to manage their schizophrenia in the way that I'd like	❍	❍	❍	❍
Time required	❍	❍	❍	❍
Travel required	❍	❍	❍	❍
Long-term commitment required	❍	❍	❍	❍
Complexity of the study logistics	❍	❍	❍	❍
Concern about loss of control	❍	❍	❍	❍
Level of compensation for participation	❍	❍	❍	❍

Part 6. General Health Information

50) In general, would you say your health is:

- Excellent
- Very Good
- Good
- Fair
- Poor

51) Compared to one year ago, how would you rate your health in general now?

- Much better now than one year ago
- Somewhat better now than one year ago
- About the same
- Somewhat worse now than one year ago
- Much worse now than one year ago

52) During the past 4 weeks, has your physical health caused any of the following problems with your work or other regular daily activities?

	Yes	No
Cut down the amount of time you spent on work or other activities	❍	❍
Accomplished less than you would like	❍	❍
Were limited in the kind of work or other activities	❍	❍
Had difficulty performing the work or other activities (for example, it took extra effort)	❍	❍

53) During the past 4 weeks, has your mental or emotional health caused any of the following problems with your work or other regular daily activities?

	Yes	No
Cut down the amount of time you spent on work or other activities	❍	❍
Accomplished less than you would like	❍	❍
Didn't do work or other activities as carefully as usual	❍	❍

54) How TRUE or FALSE is each of the following statements for you.

	1 – Definitely true	2 – Mostly true	3 - Don't know	4 – Mostly false	5 – Definitely false
I seem to get sick a little easier than other people	❍	❍	❍	❍	❍
I am as healthy as anybody I know	❍	❍	❍	❍	❍
I expect my health to get worse	❍	❍	❍	❍	❍
My health is excellent	❍	❍	❍	❍	❍

55) Have you been diagnosed with any of these medical conditions? Check all that apply.

- ☐ High Blood Pressure/Hypertension
- ☐ High Cholesterol/Hyperlipidemia
- ☐ Heart Disease/Heart Failure
- ☐ Diabetes
- ☐ Kidney Disease
- ☐ Liver Disease (Cirrhosis/Hepatitis)
- ☐ Respiratory Disease (COPD, Asthma)
- ☐ Other – Please specify: *

56) Do you currently use of any of the following? Check all that apply and try to answer honestly, knowing that all individual responses will remain confidential to our survey team.

	Never	Rarely	Sometimes	Often
Alcohol	❍	❍	❍	❍
Tobacco, including smoking	❍	❍	❍	❍
Marijuana	❍	❍	❍	❍
Prescription opioid	❍	❍	❍	❍
Any illicit substances	❍	❍	❍	❍

Part 7. Demographic Information

LOGIC

Page exit logic: Skip / Disqualify Logic IF: TRUE is always true THEN: Jump to page 19 - Thank You & Invitation to Participate in Patient-Focused Drug Development Meeting

This is the last section of our survey. It asks for information that will help us to create a more complete picture of how schizophrenia affects different people. Once again, all individual responses will remain confidential. Completion of this final section will help us better represent the schizophrenia community's experiences and perspectives.

57) With which gender do you most identify?

- ☐ Male
- ☐ Female
- ☐ Non-binary
- ☐ Another identity not listed: ______
- ☐ Prefer not to answer

58) Are you Hispanic, Latino, or of Spanish origin?

- ☐ Yes
- ☐ No
- ☐ Prefer not to answer

59) Which of the following best describe(s) you? Check all that apply.

- ☐ American Indian or Alaska Native
- ☐ Asian
- ☐ Black or African American
- ☐ Native Hawaiian or Other Pacific Islander
- ☐ White
- ☐ Another identity not listed:(please specify): ______
- ☐ Prefer not to answer

60) What is the highest level of education that you have completed?

- Some high school
- High school
- Associate's degree or equivalent
- Bachelor’s degree
- Master’s degree
- Doctoral degree
- Prefer not to answer

61) What type(s) of insurance do you have? Check all that apply.

- Employer-sponsored health insurance
- Commercial health insurance obtained through the marketplace
- Medicare
- Medicaid
- Short-term disability insurance
- Long-term disability insurance
- Other, please specify: ______
- None
- I don't know
- Prefer not to answer

Consent to Participate (Caregivers)

This form has important information about why this survey is being done, what participation involves, and how the information survey respondents provide will be used.

LOGIC

Page exit logic: Skip / Disqualify Logic IF: (#62 Question "Please check each of the boxes below to indicate your understanding of these terms and consent to participate." is not exactly equal to ("I have read this consent form, or it has been read to me. I understand the information in this form, have had an opportunity to ask questions, and have had my questions answered. If I have additional questions, I understand that I can contact the research team. I agree to participate in this study and agree that the information I provide can be used as described.","I have reached the age of majority (19 in Alabama and Nebraska, 21 in Mississippi and Puerto Rico, and 18 in all other states).","I live in the United States.","As a caregiver, I confirm that I am authorized to complete this survey on behalf of the person for whom I care and consent to the use of their information in research.") OR #65 Question "Please enter your age in years" is less than "18") THEN: Disqualify and display: "Thank you for your interest in this survey. This survey is only for individuals who have reached the age of majority, and respondents must indicate agreement with all terms listed on the prior page. If you are an individual with schizophrenia who is younger than the age of majority, we encourage you to ask your parent or legal guardian of majority age to complete the survey on your behalf. Please contact the research team if you have any questions."

What are the goals of this study?

You are being asked to participate in a research study about the experiences of an individual with schizophrenia for whom you provide care. The study's goals are to:

- Provide insight into the frequency, severity, and significance of symptoms experienced by people with the schizophrenia;
- Describe how schizophrenia symptoms impact daily life;
- Describe approaches that people with schizophrenia use to treat and manage the disease and its symptoms;
- Provide perspective on why people do or do not use medications prescribed for schizophrenia; and
- Identify desired outcomes of schizophrenia medications that might be developed in the future.

This survey was commissioned by the Schizophrenia and Psychosis Action Alliance (S&PAA) in partnership with the American Foundation for Suicide Prevention, Mental Health America, the National Alliance on Mental Illness, and the National Council for Mental Wellbeing. It is being conducted by Faegre Drinker Consulting (Faegre Drinker).

What will I do if I choose to participate?

You will be asked to complete a survey about the experiences that the person you care for has had with schizophrenia, and what you believe they would like from therapies for schizophrenia. The survey should take about 15 minutes.

What are my rights as a research participant?

Participation in this study is voluntary. If you begin the survey, you can stop at any time. You do not have to answer any question you do not want to answer. You will not be penalized if you decide to quit the survey.

How will you use the information that you collect?

This research will help patient organizations, therapy developers, and the U.S. Food and Drug Administration (FDA) better understand what matters when it comes to symptoms, impacts on daily life, and development of new therapies. Survey results will inform planning for a community-led meeting with the FDA in late 2022. Summary results will also be shared in a public report after the meeting. Results of this study also may be used in subsequent publications and presentations. If results of this study are published or presented, individual names and other personally identifiable information will be removed. We may share the data we collect from you with other researchers but will not share any of your personal identifiable information.

What benefits will I get from participating?

This study is designed to learn more about the experiences and views of those living with and caring for people with schizophrenia. Study results may be used in the future to help individuals with schizophrenia, as well as their caregivers and families, and inform U.S. Food and Drug Administration decisions about therapies for schizophrenia. You are not likely to have any direct benefits from participating.

What are the risks or discomforts associated with participation?

This survey does not involve any significant physical risks. As with all research, there is a chance that confidentiality of the information we collect about you could be breached. We take steps to minimize this risk, as described below.
Because the survey asks about experiences with schizophrenia, it is possible that some questions could be upsetting. If you find yourself in distress, help is available:

- The NAMI HelpLine—800-950-6264 or info@nami.org—can offer sympathy and support and provide information about resources in your community. To find a warmline in your area, dial 211, or go to www.211.info, for information on local social services.

- The American Foundation for Suicide Prevention provides information about suicide and preventing suicide as well as support for those affected by suicide. Reach the Crisis Text Line at crisistextline.org, call 1-800-273-8255, or text TALK to 741741 to connect with a trained crisis counselor for free, 24/7.
- The National Suicide Prevention Lifeline provides 24/7, free and confidential support for people in distress, prevention and crisis resources for you or your loved ones, and best practices for professionals. Find more information about the Lifeline at suicidepreventionlifeline.org or dial 800-273-8255.

How will the information I provide be protected?

This survey is hosted on the Alchemer platform, which encrypts data and can only be accessed through secure HTTPS. Data will be analyzed by a team of individuals from Faegre Drinker and S&PAA. All personal information collected via the survey will remain confidential to this team, and all information shared in papers or reports on this research will be de-identified. Additionally, any data stored for the long term or used in comparison to other data in future research will be de-linked from identity data. Neither S&PAA nor Faegre Drinker will sell your data. By submitting personal information through this survey or to a Faegre Drinker or S&PAA contact, you agree that Faegre Drinker and S&PAA may contact you with questions related to the survey.

Who can I contact if I have questions or concerns about this study?

If you have any questions about this study, you can contact the research team via this form. You may also contact Advarra Institutional Review Board (IRB) with questions about research participants' rights or read more here.

VALIDATION
Min. answers = 3 (if answered)

62) Please check each of the boxes below to indicate your understanding of these terms and consent to participate.*

- ☐ I have read this consent form, or it has been read to me. I understand the information in this form, have had an opportunity to ask questions, and have had my questions answered. If I have additional questions, I understand that I can contact the research team. I agree to participate in this study and agree that the information I provide can be used as described.
- ☐ I have reached the age of majority (19 in Alabama and Nebraska, 21 in Mississippi and Puerto Rico, and 18 in all other states).
- ☐ I live in the United States.
- ☐ As a caregiver, I confirm that I am authorized to complete this survey on behalf of the person for whom I care and consent to the use of their information in research.

63) Information about the Person for Whom You Care*

First Name:

Last Name:

Age in Years (Number):

64) Please sign this form by completing each of the fields below:*

First Name:

Last Name:

VALIDATION

Must be numeric Whole numbers only Positive numbers only

65) Please enter your age in years

Part 1. Initial Symptoms and Diagnosis (Caregiver Questions)

As you continue with this survey, you will be asked questions about initial schizophrenia symptoms and diagnosis, daily life, research, and disease management and treatment. Please answer on behalf of the person you care for, and try to answer as accurately as you can. If you don't know the answer to a question, it is OK to leave it blank.

VALIDATION

Must be numeric Whole numbers only Positive numbers only

66) How many years old was the person you care for when...

They first experienced symptoms of schizophrenia?:

They were officially diagnosed with schizophrenia?:

67) Before receiving a diagnosis of schizophrenia, did the person you care for experience any of the following health-related events or circumstances? Check all that apply.

- ☐ Started eating less regularly
- ☐ Had trouble sleeping
- ☐ Increased use of alcohol or drugs
- ☐ Received treatment for substance use disorder
- ☐ Had thoughts of self-harm
- ☐ Attempted suicide
- ☐ Was hospitalized for psychiatric reasons

68) Before receiving a diagnosis of schizophrenia, did the person you care for experience or do any of the following life events or circumstances? Check all that apply.

- Struggled with relationships
- Had difficulty doing their job
- Lost their job
- Had difficulty in school
- Was expelled from school
- Was the victim of a crime or abuse
- Experienced homelessness
- Was arrested because of behaviors related to schizophrenia
- Was incarcerated in prison or jail
- Needed legal services not related to being arrested (for example, help with legal guardianship, disability rights, other civil matters)
- Needed increased special education or employment support services

69) Around the time the person you care for received a diagnosis of schizophrenia, did they experience any of the following? Check all that apply.

- Thoughts or fears related to cleanliness and contamination
- Difficulty tolerating uncertainty or disorganization
- Thoughts about harming themselves or others
- Repetitive behaviors such as frequent washing, cleaning, counting, or demanding reassurance
- Loss of motivation
- Loss of interest in daily activities
- Loss of interest in social activities
- Lack of energy
- Seeing, hearing, tasting, smelling, or feeling things that others did not
- Feeling convinced that you were extremely special or talented
- Experiences with telepathy, psychic forces, or fortune telling
- Feeling more afraid than usual
- Feeling they were being watched
- Not trusting other people
- Believing things others didn't believe

70) Around the time the person you care for received a diagnosis of schizophrenia, did they have difficulty with any of the following? Check all that apply.

- Expressing emotions
- Controlling emotions
- Speaking clearly so that others can understand them
- Thinking clearly
- Relating to others
- Focusing on conversations
- Concentrating on tasks
- Remembering things
- Learning new information
- Solving problems
- Making decisions
- Taking care of themselves
- Working
- Starting and/or finishing things

71) Have any relatives of the person you care for been diagnosed with schizophrenia or a related condition? If so, please check the box(es) for anyone who has.

- No, not to my knowledge
- Brother or sister
- Cousin
- Parent
- Aunt or uncle
- Grandparent
- Niece or Nephew
- Other – Please Describe: ____________

72) Before the person you care for was diagnosed with schizophrenia, did they receive any other diagnoses to explain the symptoms that they were experiencing?

- Yes
- No

LOGIC

Logic: Hidden unless: #10 Question "Before you were diagnosed with schizophrenia, did you receive any other diagnoses to explain the symptoms that you were experiencing?" is one of the following answers ("Yes")

73) Did they receive any of the following diagnoses? Check all that apply.

- Anxiety (e.g., generalized anxiety disorder, social anxiety disorder, phobias)
- Attention deficit hyperactivity disorder (ADHD)
- Autism Spectrum Disorder
- Bipolar disorder
- Major depressive disorder (MDD)
- Post-traumatic stress disorder (PTSD)
- Borderline personality disorder
- Traumatic brain injury (TBI)
- Delusional disorder
- Obsessive compulsive disorder (OCD)
- Schizoaffective disorder
- Schizophreniform disorder
- Substance use disorder
- Other Diagnosis - Please Specify: __________

74) In addition to schizophrenia, does the person you care for currently have an official diagnosis of any of the following? Check all that apply.

- Anxiety, including generalized anxiety disorder, social anxiety disorder, and phobias
- Attention deficit hyperactivity disorder (ADHD)
- Autism Spectrum Disorder
- Bipolar disorder
- Borderline personality disorder
- Delusional disorder
- Major depressive disorder (MDD)
- Obsessive compulsive disorder
- Post-traumatic stress disorder (PTSD)
- Schizoaffective disorder
- Schizophreniform disorder
- Substance use disorder
- Traumatic brain injury (TBI)
- Other Diagnosis - Please Specify: __________

Part 2. Living with Schizophrenia: Symptoms (Caregiver Questions)

75) Schizophrenia may make it difficult to think, act, or live the way one wants. Have any of the following diminished the ability of the person you care for to function and live the way they would like?

	No Impact	Minimal Impact	Moderate Impact	Significant Impact
Thoughts or fears related to cleanliness and contamination	❍	❍	❍	❍
Difficulty tolerating uncertainty or disorganization	❍	❍	❍	❍
Thoughts about losing control and harming yourself or others	❍	❍	❍	❍
Repetitive behaviors such as frequent washing, cleaning, counting, or demanding reassurance	❍	❍	❍	❍
Loss of motivation	❍	❍	❍	❍
Loss of interest in daily activities	❍	❍	❍	❍
Loss of interest in social activities	❍	❍	❍	❍
Lack of energy	❍	❍	❍	❍
Seeing, hearing, tasting, smelling, or feeling things that others do not	❍	❍	❍	❍
Feeling convinced that you were extremely special or talented	❍	❍	❍	❍
Experiences with telepathy, psychic forces, or fortune telling	❍	❍	❍	❍
Feeling more afraid than usual	❍	❍	❍	❍
Feelings of being watched	❍	❍	❍	❍
Not trusting other people	❍	❍	❍	❍
Believing something that others don’t believe	❍	❍	❍	❍

76) In the past six months, has the person you care for had difficulty with any of the following?

	No Difficulty	Minimal Difficulty	Moderate Difficulty	Significant Difficulty
Expressing emotions	❍	❍	❍	❍
Controlling emotions	❍	❍	❍	❍
Speaking so that others can understand me	❍	❍	❍	❍
Thinking clearly	❍	❍	❍	❍
Relating to others	❍	❍	❍	❍
Focusing on conversations	❍	❍	❍	❍
Concentrating on tasks	❍	❍	❍	❍
Remembering things	❍	❍	❍	❍
Learning new information	❍	❍	❍	❍
Solving problems	❍	❍	❍	❍
Making decisions	❍	❍	❍	❍
Taking care of yourself	❍	❍	❍	❍
Working	❍	❍	❍	❍
Starting and/or finishing things	❍	❍	❍	❍

77) In the past 12 months, has the person you care for done or experienced any of the following? Check all that apply. Please remember that all individual responses will remain confidential, and honest answers will help ensure that we are accurately representing the schizophrenia community.

- ☐ Received treatment for substance use disorder
- ☐ Been the victim of a crime or abuse
- ☐ Been hospitalized for psychiatric reasons
- ☐ Had thoughts of self-harm
- ☐ Attempted suicide
- ☐ Experienced homelessness
- ☐ Been arrested
- ☐ Been incarcerated in prison or jail
- ☐ Needed other legal services (including but not limited to legal guardianship, disability rights, other civil matters)
- ☐ Needed special education or employment support services beyond their usual needs
- ☐ Overdosed on drugs
- ☐ Other - Write In: ____________________

78) How well do the below statements describe the person you care for and what they think about their experience today?

	Agree Very Much	Agree	Unsure	Disagree	Disagree very much
Some of the symptoms were made up by their mind	❍	❍	❍	❍	❍
They do not need medication	❍	❍	❍	❍	❍
The doctor is right in prescribing medication for them	❍	❍	❍	❍	❍
If someone told them that I had a mental illness they would be right	❍	❍	❍	❍	❍
Their stay in the hospital was necessary	❍	❍	❍	❍	❍
None of the unusual things they experienced are due to an illness	❍	❍	❍	❍	❍
They do not need to be seen by a doctor or psychiatrist	❍	❍	❍	❍	❍
They are mentally well	❍	❍	❍	❍	❍

VALIDATION

Must be numeric

79) From the time the person you care for received their schizophrenia diagnosis to today, about how many times have they experienced a setback or "recurrence" in their symptoms?

80) How severe have the schizophrenia symptoms of the person you care for been...

	No symptoms	Mild	Moderate	Severe
During the past week	❍	❍	❍	❍
During the past 30 days	❍	❍	❍	❍

81) How much do schizophrenia symptoms negatively impact the life of the person you care for...

	No Impact	Minimal Impact	Moderate Impact	Significant Impact
On the best days	❍	❍	❍	❍
On the worst days	❍	❍	❍	❍

VALIDATION

Max character count = 100

82) What have been the most significant impacts of schizophrenia on their life?

Part 3. Living with Schizophrenia: Daily Life (Caregiver Questions)

83) Which of these best describes the current living situation of the person for whom you care?

- Living independently, alone
- A household with relatives or friends
- Supervised group housing
- Partially supervised group housing
- Residential substance use program (e.g., sober house, halfway house)
- Supportive housing
- Long-term care facility, such as a nursing home, senior housing, or a skilled nursing facility
- Correctional facility (e.g., prison, jail)
- Shelter
- Currently without housing
- I don't know

84) Has the person you care for had any other living situations within the past 12 months? Check all that apply.

- Living independently, alone
- A household with relatives or friends
- Supervised group housing
- Partially supervised group housing
- Residential substance use program (e.g., sober house, halfway house)
- Supportive housing
- Long-term care facility, such as a nursing home, senior housing, or a skilled nursing facility
- Correctional facility (e.g., prison, jail)
- Shelter
- Currently without housing
- I don't know

85) Is the person you care for currently employed, or working for pay?

- Yes, full-time
- Yes, part-time
- Actively looking for work but not currently employed
- Not currently employed
- Retired
- Prefer not to say

86) Is the person you care for currently in school?

- Yes, full-time
- Yes, part-time
- No
- I don't know

87) Since the person you care for first started experiencing symptoms of schizophrenia, has their ability to do the following changed?

	Worsened significantly	Worsened	No change	Improved	Improved significantly	Not applicable
Manage relationships	❍	❍	❍	❍	❍	❍
Perform in their job	❍	❍	❍	❍	❍	❍
Type of job that they can hold	❍	❍	❍	❍	❍	❍
Perform in school	❍	❍	❍	❍	❍	❍
Manage their responsibilities at home	❍	❍	❍	❍	❍	❍
Manage their finances	❍	❍	❍	❍	❍	❍
Cope without alcohol or drugs	❍	❍	❍	❍	❍	❍

88) Has the person you care for made any of the following changes in their career or education because of schizophrenia symptoms? Check all that apply.

- Cut back on hours at work
- Took a leave of absence from a job
- Took a leave of absence from school
- Switched to a job with fewer or more predictable hours
- Switched to a less challenging job
- Switched to a job that they were overqualified for
- Passed up a promotion or desired job change
- Postponed or cancelled future education plans
- Left the workforce altogether
- Took early retirement
- Other - Write In: ____________

89) Has the person you care for done any of the following because of schizophrenia symptoms? Check all that apply.

- Avoided important social gatherings
- Stopped pursuing hobbies
- Avoided taking vacation
- Ended a romantic relationship
- None of the above
- Other - Write In: ____________________

90) Has the person you care for ever used or taken part in any of the following forms of support? Check all that apply.

- Financial support from family or friends
- Financial assistance from the government
- Disability insurance
- Supported employment
- Vocational rehabilitation other than supported employment
- Clubhouse program
- Respite care
- Other - Write In: ____________________

91) Has the person you care for ever had a psychiatric advance directive (PAD) in place? This could include a conservatorship/guardianship, medical or mental health power of attorney, financial power of attorney, or court-mandated treatment.

- Yes
- No
- I don't know

LOGIC

Hidden unless: #29 Question "Have you ever had a psychiatric advance directive (PAD) in place? This could include a conservatorship/guardianship, medical or mental health power of attorney, financial power of attorney, or court-mandated treatment." is one of the following answers ("Yes")

92) Have they ever been under court-mandated treatment or conservatorship?

- Yes
- No
- I don't know

Part 4. Treating and Managing Schizophrenia (Caregiver Questions)

93) When thinking about new treatments for schizophrenia, how important to them would it be that treatments...

	Not important	Minimally important	Moderately important	Very important
Prevent delusions	❍	❍	❍	❍
Prevent hallucinations	❍	❍	❍	❍
Improve motivation	❍	❍	❍	❍
Help them feel energized	❍	❍	❍	❍
Help them enjoy daily activities	❍	❍	❍	❍
Make it easier to maintain relationships	❍	❍	❍	❍
Make decision-making easier	❍	❍	❍	❍
Improve their ability to pay attention	❍	❍	❍	❍
Improve their concentration	❍	❍	❍	❍
Improve their memory	❍	❍	❍	❍
Prevent involuntary movements	❍	❍	❍	❍
Reduce agitation	❍	❍	❍	❍

94) About how many hours per week does the person you care for spend actively managing their schizophrenia? This can include time spent attending medical or therapy appointments, traveling to and from appointments, and spending time on their own dealing with or trying to improve symptoms.

- 0–5 hours
- 5–10 hours
- 10–15 hours
- 15–20 hours
- More than 20 hours

95) What types of non-drug treatment or support has the person you care for received? Are they receiving these currently?

	In the past	Currently
Inpatient psychiatric treatment	❍	❍
Substance use disorder treatment	❍	❍
Outpatient psychiatric or psychological treatment (therapy, counseling, or support groups)	❍	❍
Housing support services	❍	❍
Employment or education support services	❍	❍
None of the above	❍	❍

96) Is the person you care for currently taking any medications to manage their schizophrenia? Check all that apply.

- Oral antipsychotic medication
- Long-acting injectable antipsychotic medication
- Other psychiatric medication (such as antidepressants or anxiolytics)
- I don't know
- Other - Write In:

LOGIC

Hidden unless: #34 Question "Are you currently taking any medications to manage your schizophrenia? Check all that apply." is one of the following answers ("Oral antipsychotic medication","Long-acting injectable antipsychotic medication","Other psychiatric medication (such as antidepressants or anxiolytics)")

97) Does the person you care for take their medication(s) as prescribed by their doctor?

- Yes
- No
- I don't know

VALIDATION

Must be numeric

98) How many different schizophrenia medications has the person you care for taken since they were diagnosed with schizophrenia?

99) Has the person you care for ever switched from one medication to another for the reasons below? Check all that apply.

- A medication that they were taking did not effectively treat their symptoms
- Their doctor recommended taking a different medication
- A medication that they were taking had undesirable side effects
- Their insurance coverage for the new medication was better
- Their insurance stopped covering the medication they were taking
- Other - Write In:

100) Has the person you care for ever quit taking a medication prescribed to treat their schizophrenia for any of the following reasons? Check all that apply.

- ▯ Their medication regimen was too complicated
- ▯ It did not treat their symptoms
- ▯ It caused them to feel flat
- ▯ It caused them to gain weight
- ▯ It caused movement effects (tremor, stiffness, agitation)
- ▯ It caused sexual problems
- ▯ It prompted loss of their menstrual period
- ▯ It caused breast lactation
- ▯ It was not covered by their insurance
- ▯ Their symptoms resolved
- ▯ Other - Write In: ____________________

Part 5. Research Studies (Caregiver Questions)

101) Has the person you care for participated in any of the following types of research studies? Check all that apply. If you don't know, select "I don't know."

- ▯ Natural history study for schizophrenia – These studies seek to understand how diseases progress or change over time, without treatment.
- ▯ Other observational study for schizophrenia – These include other studies that assess health outcomes but do not involve specific interventions, such as changes in diet, exercise, or use of medications or devices
- ▯ Interventional study for schizophrenia that tested the effects of something other than a drug – Examples of interventional studies that are not for investigational drugs include studies for which participants might change their diets, exercise habits, or other activities.
- ▯ I don't know

LOGIC

Show/hide trigger exists.

102) Has the person you care for ever participated in a clinical trial for an investigational drug for schizophrenia? These studies test the safety and efficacy of medications to determine whether they should be approved for use outside of research studies.

- ▯ Yes
- ▯ No
- ▯ I don't know

LOGIC

Hidden unless: #102 Question "Has the person you care for ever participated in a clinical trial for an investigational drug for schizophrenia? These studies test the safety and efficacy of medications to determine whether they should be approved for use outside of research studies." is one of the following answers ("Yes")

103) How much motivation, if any, did the following provide to participate in the trial?

	Not at all	Minimal Motivation	Moderate Motivation	Significant Motivation
Desire for clinical benefit and improved quality of life	❍	❍	❍	❍
Access to investigational drug	❍	❍	❍	❍
Access to medical care	❍	❍	❍	❍
Access to medical experts	❍	❍	❍	❍
Positive reputation of the study team	❍	❍	❍	❍
Positive interactions with the study team	❍	❍	❍	❍
Opportunity to help others with schizophrenia	❍	❍	❍	❍
Opportunity to contribute to science	❍	❍	❍	❍
Support from their doctor	❍	❍	❍	❍
Suggested by another person whose family is impacted by the disease	❍	❍	❍	❍
Suggested by a patient advocacy organization	❍	❍	❍	❍

LOGIC

Hidden unless: #102 Question "Has the person you care for ever participated in a clinical trial for an investigational drug for schizophrenia? These studies test the safety and efficacy of medications to determine whether they should be approved for use outside of research studies." is one of the following answers ("Yes")

104) Did any of the following cause concern about participation in the trial? Check all that apply.

- ▯ Concern about not being accepted into the trial
- ▯ Uncertainty about the safety of the study drug
- ▯ Uncertainty about whether potential benefits would justify potential risks
- ▯ Fear of receiving placebo
- ▯ Fear of physical and/or mental pain that could accompany required tests
- ▯ Uncertainty about whether the study requirements would allow me to manage their schizophrenia in the way that I'd like
- ▯ Time required
- ▯ Travel required
- ▯ Long-term commitment required
- ▯ Complexity of the study logistics
- ▯ Concern about loss of control
- ▯ Level of compensation for participation
- ▯ Other - Write In: ____________________

LOGIC

Show/hide trigger exists. Hidden unless: #102 Question "Has the person you care for ever participated in a clinical trial for an investigational drug for schizophrenia? These studies test the safety and efficacy of medications to determine whether they should be approved for use outside of research studies." is one of the following answers ("Yes")

105) Would the person you care for consider joining a clinical trial again?

- ▯ Yes
- ▯ No
- ▯ I don't know

LOGIC

Hidden unless: #105 Question "Would the person you care for consider joining a clinical trial again?" is one of the following answers ("No")

106) Why not?

LOGIC

Hidden unless: #102 Question "Has the person you care for ever participated in a clinical trial for an investigational drug for schizophrenia? These studies test the safety and efficacy of medications to determine whether they should be approved for use outside of research studies." is one of the following answers ("No")

107) Before taking this survey, were you aware that there are research studies on new medications that haven't yet been proven to be effective but might be?

- Yes
- No
- I don't know

LOGIC

Hidden unless: #102 Question "Has the person you care for ever participated in a clinical trial for an investigational drug for schizophrenia? These studies test the safety and efficacy of medications to determine whether they should be approved for use outside of research studies." is one of the following answers ("No")

108) Has the person you care for ever tried to participate in a clinical trial but have not been eligible?

- Yes
- No
- I don't know

LOGIC

Hidden unless: #102 Question "Has the person you care for ever participated in a clinical trial for an investigational drug for schizophrenia? These studies test the safety and efficacy of medications to determine whether they should be approved for use outside of research studies." is one of the following answers ("No")

109) Would the person you care for consider joining a clinical trial in the future?

- Yes
- No
- I don't know

LOGIC

Hidden unless: #102 Question "Has the person you care for ever participated in a clinical trial for an investigational drug for schizophrenia? These studies test the safety and efficacy of medications to determine whether they should be approved for use outside of research studies." is one of the following answers ("No","I don't know")

110) If the person you care for were given the chance to participate in a clinical trial, how much motivation do you think the following would provide to participate?

	Not at all	Minimal Motivation	Moderate Motivation	Significant Motivation
Desire for clinical benefit and improved quality of life	❍	❍	❍	❍
Access to investigational drug	❍	❍	❍	❍
Access to medical care	❍	❍	❍	❍
Access to medical experts	❍	❍	❍	❍
Positive reputation of the study team	❍	❍	❍	❍
Positive interactions with the study team	❍	❍	❍	❍
Opportunity to help others with schizophrenia	❍	❍	❍	❍
Opportunity to contribute to science	❍	❍	❍	❍
Support from their doctor	❍	❍	❍	❍
Suggested by another person whose family is impacted by the disease	❍	❍	❍	❍
Suggested by a patient advocacy organization	❍	❍	❍	❍

LOGIC

Hidden unless: #102 Question "Has the person you care for ever participated in a clinical trial for an investigational drug for schizophrenia? These studies test the safety and efficacy of medications to determine whether they should be approved for use outside of research studies." is one of the following answers ("No","I don't know")

111) If the person you care for were given the chance to participate in a clinical trial in the future, how much concern do you think the following might cause them?

	No Concern	Minimal Concern	Moderate Concern	Significant Concern
Concern about not being accepted into the trial	❍	❍	❍	❍
Uncertainty about the safety of the study drug	❍	❍	❍	❍
Uncertainty about whether potential benefits would justify potential risks	❍	❍	❍	❍
Fear of receiving placebo	❍	❍	❍	❍
Fear of physical and/or mental pain that could accompany required tests	❍	❍	❍	❍
Uncertainty about whether the study requirements would allow me to manage their schizophrenia in the way that I'd like	❍	❍	❍	❍

	No Concern	Minimal Concern	Moderate Concern	Significant Concern
Time required	❍	❍	❍	❍
Travel required	❍	❍	❍	❍
Long-term commitment required	❍	❍	❍	❍
Complexity of the study logistics	❍	❍	❍	❍
Concern about loss of control	❍	❍	❍	❍
Level of compensation for participation	❍	❍	❍	❍

Part 6. General Health Information (Caregiver Questions)

112) In general, would you say health of the person you care for is:

- Excellent
- Very Good
- Good
- Fair
- Poor

113) Compared to one year ago, how would you rate the health of the person you care for in general now?

- Much better now than one year ago
- Somewhat better now than one year ago
- About the same
- Somewhat worse now than one year ago
- Much worse now than one year ago

114) During the past 4 weeks, has the physical health of the person you care for caused any of the following problems with their work or other regular daily activities?

	Yes	No
Cut down the amount of time they spent on work or other activities	❍	❍
Accomplished less than they would like	❍	❍
Were limited in the kind of work or other activities	❍	❍
Had difficulty performing the work or other activities (for example, it took extra effort)	❍	❍

115) During the past 4 weeks, has the mental or emotional health of the person you care for caused any of the following problems with their work or other regular daily activities?

	Yes	No
Cut down the amount of time they spent on work or other activities	❍	❍
Accomplished less than they would like	❍	❍
Didn't do work or other activities as carefully as usual	❍	❍

116) How TRUE or FALSE is each of the following statements for the person for whom you care.

	1 – Definitely true	2 – Mostly true	3 - Don't know	4 – Mostly false	5 – Definitely false
They seem to get sick a little easier than other people	❍	❍	❍	❍	❍
They are as healthy as anybody they know	❍	❍	❍	❍	❍
They expect their health to get worse	❍	❍	❍	❍	❍
Their health is excellent	❍	❍	❍	❍	❍

117) Has the person you care for been diagnosed with any of these medical conditions? Check all that apply.

- ▯ High Blood Pressure/Hypertension
- ▯ High Cholesterol/Hyperlipidemia
- ▯ Heart Disease/Heart Failure
- ▯ Diabetes
- ▯ Kidney Disease
- ▯ Liver Disease (Cirrhosis/Hepatitis)
- ▯ Respiratory Disease (COPD, Asthma)
- ▯ Other – Please specify: * ____________________

118) Does the person you care for currently use of any of the following? Check all that apply and try to answer honestly, knowing that all individual responses will remain confidential to our survey team.

	Never	Rarely	Sometimes	Often	I don't know
Alcohol	❍	❍	❍	❍	❍
Tobacco, including smoking	❍	❍	❍	❍	❍
Marijuana	❍	❍	❍	❍	❍
Prescription opioid	❍	❍	❍	❍	❍
Any illicit substances	❍	❍	❍	❍	❍

Part 7. Demographic Information (Caregiver Questions)

LOGIC

Page exit logic: Skip / Disqualify Logic IF: TRUE is always true THEN: Jump to page 19 - Thank You & Invitation to Participate in Patient-Focused Drug Development Meeting

This is the last section of our survey. It asks for information that will help us to create a more complete picture of how schizophrenia affects different people. Once again, all individual responses will remain confidential. Completion of this final section will help us better represent the schizophrenia community's experiences and perspectives. Please complete the below fields for the person for whom you care.

119) With which gender does the person you care for most identify with?

- ☐ Male
- ☐ Female
- ☐ Non-binary
- ☐ Another identity not listed: ______
- ☐ Prefer not to answer

120) Is the person you care for Hispanic, Latino, or of Spanish origin?

- ☐ Yes
- ☐ No
- ☐ Prefer not to answer

121) Which of the following best describe(s) the person for whom you care? Check all that apply.

- ☐ American Indian or Alaska Native
- ☐ Asian
- ☐ Black or African American
- ☐ Native Hawaiian or Other Pacific Islander
- ☐ White
- ☐ Another identity not listed:(please specify): ______
- ☐ Prefer not to answer

122) What is the highest level of education the person you care for has completed?

- ▯ Some high school
- ▯ High school
- ▯ Associate's degree or equivalent
- ▯ Bachelor’s degree
- ▯ Master’s degree
- ▯ Doctoral degree
- ▯ I don't know
- ▯ Prefer not to answer

123) What type(s) of insurance does the person you care for have? Check all that apply.

- ▯ Employer-sponsored health insurance
- ▯ Commercial health insurance obtained through the marketplace
- ▯ Medicare
- ▯ Medicaid
- ▯ Short-term disability insurance
- ▯ Long-term disability insurance
- ▯ Other, please specify: ____________
- ▯ None
- ▯ I don't know
- ▯ Prefer not to answer

Thank You & Invitation to Participate in Patient-Focused Drug Development Meeting

We appreciate your participation in this research. The information you've shared about your experiences and attitudes will help our efforts to advance therapy development for schizophrenia and address unmet needs within our community. Before you go, we'd love to hear just a few more things.

124) Would you like to receive an email when the results of this survey are published?

- Yes
- No

125) Are you interested in attending the Fall 2022 Schizophrenia Patient-Focused Drug Development meeting in the Washington, DC area?

- Yes, and I'd be willing to serve as a panelist
- Yes, but I do not want a speaking role
- No

126) How did you first hear about this survey?

- Advocacy organization website: [Enter name]:
- Doctor or other healthcare provider
- Friend or family member
- Schizophrenia PFDD Meeting website
- Other Internet site or social media
- Flyer or other printed material
- Other (please describe):*

127) Would you like to receive future information about other research opportunities from the Schizophrenia and Psychosis Action Alliance and its partners?

- Yes
- No

128) If you are interested in receiving information from us in the future or indicated interest in the Patient-Focused Drug Development meeting, what is your preferred email address?

If you would like to attend the Schizophrenia Fall 2022 Patient-Focused Drug Development Meeting in the Washington, DC area live or via the live webcast, you can register here. If you indicated interest in speaking on a panel, we will be in touch in the coming weeks.

Confirmation of Submission

Thank you for taking our survey. We've recorded your responses, and look forward to being able to share the results of this survey.
